# Promising potential of [^177^Lu]Lu-DOTA-folate to enhance tumor response to immunotherapy—a preclinical study using a syngeneic breast cancer model

**DOI:** 10.1007/s00259-020-05054-9

**Published:** 2020-10-19

**Authors:** Patrycja Guzik, Klaudia Siwowska, Hsin-Yu Fang, Susan Cohrs, Peter Bernhardt, Roger Schibli, Cristina Müller

**Affiliations:** 1 Center for Radiopharmaceutical Sciences ETH-PSI-USZ, Paul Scherrer Institute, 5232 Villigen-PSI, Switzerland; 2 Department of Radiation Physics, The Sahlgrenska Academy, University of Gothenburg, SE-413 45 Gothenburg, Sweden; 3 Department of Medical Physics and Medical Bioengineering, Sahlgrenska University Hospital, SE-413 45 Gothenburg, Sweden; 4 Department of Chemistry and Applied Biosciences, ETH Zurich, 8093 Zurich, Switzerland

**Keywords:** Folate receptor, Immunotherapy, NF9006 breast tumor cells, CTLA-4, [^177^Lu]Lu-DOTA-folate

## Abstract

**Purpose:**

It was previously demonstrated that radiation effects can enhance the therapy outcome of immune checkpoint inhibitors. In this study, a syngeneic breast tumor mouse model was used to investigate the effect of [^177^Lu]Lu-DOTA-folate as an immune stimulus to enhance anti-CTLA-4 immunotherapy**.**

**Methods:**

In vitro and in vivo studies were performed to characterize NF9006 breast tumor cells with regard to folate receptor (FR) expression and the possibility of tumor targeting using [^177^Lu]Lu-DOTA-folate. A preclinical therapy study was performed over 70 days with NF9006 tumor-bearing mice that received vehicle only (group A); [^177^Lu]Lu-DOTA-folate (5 MBq; 3.5 Gy absorbed tumor dose; group B); anti-CTLA-4 antibody (3 × 200 μg; group C), or both agents (group D). The mice were monitored regarding tumor growth over time and signs indicating adverse events of the treatment.

**Results:**

[^177^Lu]Lu-DOTA-folate bound specifically to NF9006 tumor cells and tissue in vitro and accumulated in NF9006 tumors in vivo. The treatment with [^177^Lu]Lu-DOTA-folate or an anti-CTLA-4 antibody had only a minor effect on NF9006 tumor growth and did not substantially increase the median survival time of mice (23 day and 19 days, respectively) as compared with untreated controls (12 days). [^177^Lu]Lu-DOTA-folate sensitized, however, the tumors to anti-CTLA-4 immunotherapy, which became obvious by reduced tumor growth and, hence, a significantly improved median survival time of mice (> 70 days). No obvious signs of adverse effects were observed in treated mice as compared with untreated controls.

**Conclusion:**

Application of [^177^Lu]Lu-DOTA-folate had a positive effect on the therapy outcome of anti-CTLA-4 immunotherapy. The results of this study may open new perspectives for future clinical translation of folate radioconjugates.

**Electronic supplementary material:**

The online version of this article (10.1007/s00259-020-05054-9) contains supplementary material, which is available to authorized users.

## Introduction

Immune checkpoint inhibitors (ICIs) have revolutionized cancer therapy and, thus, attracted increasing interest of clinicians over the last years [1, 2]. Immune checkpoints, including the cytotoxic T-lymphocyte antigen 4 (CTLA-4), provide inhibitory signals, which inactivate cytotoxic CD8+ T cells that are a key player in the anti-cancer immune response. ICIs such as anti-CTLA-4 antibodies are used to block these signals and, hence, stimulate the elimination of cancer cells [3, 4]. It was, however, observed that only a subset of patients responded to ICI monotherapy [2, 5]. As demonstrated in (pre)clinical studies [6, 7], the lack of response may have been ascribed to specific characteristics of the tumor, which defines it as poorly immunogenic (“cold”). In current clinical therapy settings, ICIs are, therefore, often combined with immune sensitizers, including chemotherapeutics [5, 8].

Radiation therapy has long been regarded as an exclusively genotoxic therapy modality that induces various types of cell damage and death, while radiation effects involving the immune system have been neglected for decades [9]. The discovery of the “abscopal” effect, referring to systemic anti-tumor radiation effects outside irradiated lesions, has, however, proven the impact of radiation on the tumor microenvironment [10]. Based on these observations, the rationale arose for using radiation stimuli to convert immunologically “cold” tumors into highly immunogenic (“hot”) tumors which are of vital interest to enhance the response rate to immunotherapies [9].

Breast cancer is the most frequently diagnosed cancer in women of the Western world and associated with a high mortality rate [11]. It presents mostly as systemic malignancy requiring—in addition to surgery and local treatments—chemotherapy and hormonal therapy which are, however, not always sufficiently effective [12, 13]. ICI therapy emerged as a valid alternative for the treatment of breast cancer even though the results of early clinical trials performed with ICI monotherapy in metastatic disease were modest [14]. The concept of using external radiation to sensitize poorly immunogenic tumors and enable immune response to CTLA-4 blockade has been demonstrated previously in a 4T1 breast tumor model and in several other preclinical and clinical studies [7, 15, 16]. The combination of ICI with external radiation in metastatic disease is, however, dependent on the abscopal effect to enable also the response of non-irradiated distant lesions [17].

Systemic radiation, through the use of tumor-targeted radiopharmaceuticals, seemed even more intriguing to be combined with ICI [18, 19], as it would allow reaching even smallest lesions in disseminated disease [20]. The clinical application of radiopharmaceuticals for this purpose has been scarcely described in the literature, yet several clinical studies designed to realize this promising concept are currently on going. They are aimed at investigating the beneficial effect of combining [^177^Lu]Lu-PSMA-617 or [^177^Lu]Lu-DOTATATE with ICIs for the treatment of metastasized castration-resistant prostate cancer (NCT03658447; NCT03805594) and neuroendocrine tumors (NCT03457948) as well as Merkel cell carcinomas (NCT04261855), respectively.

Tumor targeting with folate-based radioconjugates has been extensively investigated over the last two decades in (pre)clinical studies [21, 22]. More recently, folate-based radiopharmaceuticals have gained renewed interest for imaging purposes but also for therapeutic application [23–25]. The utilization of folate radioconjugates to trigger the immune response would be an additional, highly promising approach applicable for many cancer diseases due to the frequent expression of the FR on various tumor types [26–28]. In breast cancer, the FR is expressed in ~ 50% of the cases and was shown to be associated with ~ 70% of triple-negative breast cancer, which is particularly aggressive [29–33].

In this study, we evaluated and applied a murine breast cancer cell line, NF9006, originally derived from a transgenic mouse model [34, 35]. In vitro and in vivo studies were performed to investigate the possibility of targeting NF9006 tumor cells with [^177^Lu]Lu-DOTA-folate. In a proof-of-concept study, [^177^Lu]Lu-DOTA-folate was applied to NF9006 tumor-bearing mice to investigate whether this radiation stimulus would have an impact on the efficacy of anti-CTLA-4 immunotherapy.

## Materials and methods

### Radiosynthesis of the [^177^Lu]Lu-DOTA-folate

In this study, an albumin-binding DOTA-folate conjugate was used for labeling with lutetium-177 [23, 25]. The radiosynthesis was performed under standard labeling conditions at pH ~ 4.5 using no-carrier-added lutetium-177 (Medical Isotopes ITM GmbH, Germany) as previously reported (Supplementary Material Fig. S1) [25].

### Tumor cell culture

NF9006 tumor cells [35–39], a breast cancer cell line derived from MMTV-Neu transgenic mice with the FVB/N genetic background (FVB/N-Tg(MMTVneu)202Mul/J [40, 41]), were kindly provided by Prof. Martin Pruschy, University Hospital Zurich, Switzerland. KB cells (human cervical carcinoma cell line, German Collection of Microorganisms and Cell Cultures GmbH, ACC-136) and 4T1 tumor cells (kindly provided by Dr. Dyvia Vats, ETH Zurich, Switzerland) were used as FR-positive and FR-negative controls, respectively [42, 43]. NF9006 and KB cells were cultured using folate-deficient RPMI (FFRPMI) medium supplemented with 10% fetal calf serum, l-glutamine, and antibiotics and 4T1 cells were cultured in supplemented RPMI medium.

### Western blot

Western blot analysis was performed as previously reported [42] using a primary rabbit antibody against the FR (Abcam, ab67422, 1:1800) and a secondary anti-rabbit goat IgG antibody functionalized with horseradish peroxidase (Cell Signaling, 7074S, 1:3000) for detection using Amersham enhanced chemiluminescence (ECL) substrate (Prime Western Blotting Detection Reagent, GE Healthcare) (Supplementary Material). Detection of GAPDH served as a loading control (Cell Signaling, 5174S, rabbit mAb, 1:2000 and (HRP)-conjugated anti-rabbit IgG, 7074S, 1:5000). The signals were quantified to estimate the FR expression level on NF9006 tumor cells relative to KB tumor cells using ImageJ software (version 1.52d) (Supplementary Material).

### Cell experiments

The NF9006 tumor cells were used to demonstrate in vitro uptake and internalization and to determine the FR-binding affinity (K_D_ value) of [^177^Lu]Lu-DOTA-folate (25 MBq/nmol and 20 MBq/nmol, respectively) according to previously reported procedures (Supplementary Material) [25]. In vitro cell saturation experiments were performed to estimate the FR expression level in NF9006 tumor cells relative to the expression level in KB tumor cells (Supplementary Material).

### In vitro autoradiography

Autoradiography studies were performed on frozen tissue sections of NF9006, KB, and 4T1 tumors as previously reported (Supplementary Material) [44]. The sections were exposed to [^177^Lu]Lu-DOTA-folate (0.5 MBq/mL; 0.01 nmol/mL). Excess folic acid (100 μM) was added to the solution to block the FRs. After incubation, the tissue sections were washed and air-dried. Images were obtained using a storage phosphor system (Cyclone Plus, Perkin Elmer) and quantified using OptiQuant software (version 5.0, Bright Instrument Co Ltd., Perkin Elmer™).

### In vivo experiments

All applicable international, national, and institutional guidelines for the care and use of animals were followed, and the experiments were carried out according to the guidelines of Swiss Regulations for Animal Welfare. The preclinical studies were ethically approved by the Cantonal Committee of Animal Experimentation and permitted by the responsible cantonal authorities (license nos. 75679, 75721, and 79692).

Female FVB/NCrl mice were obtained from Charles River Laboratories (Sulzfeld, Germany) at the age of 6–7 weeks and fed with a folate-deficient rodent diet (ssniff Spezialdiäten GmbH; Soest, Germany). After acclimatization for 5–7 days, the mice were subcutaneously inoculated with 2.5 × 10^6^ NF9006 tumor cells in 100 μL PBS.

### Biodistribution and dosimetry of [^177^Lu]Lu-DOTA-folate

Biodistribution studies were performed in quadruplicate, 12–14 days after NF9006 tumor cell inoculation (tumor volume: ~ 100–300 mm^3^). Mice were intravenously injected with [^177^Lu]Lu-DOTA-folate (3 MBq, 0.5 nmol, 100 μL) and sacrificed at defined time points. FR-specific uptake of [^177^Lu]Lu-DOTA-folate at 4 h p.i. was confirmed by pre-injection of excess folic acid (100 μg, 100 μL per mouse) to block FRs. Selected tissues and organs were collected, weighed, and counted using a γ-counter (Perkin Elmer, Wallac Wizard 1480). The results were listed as a percentage of the injected activity per gram of tissue mass (% IA/g).

Dosimetric calculations were performed based on non-decay-corrected biodistribution data (Supplementary Material Table S1). The cumulated activity was estimated by calculating the time-integrated activity concentration coefficients (TIACCs) and used for calculation of the mean specific absorbed dose (Gy/MBq) to the NF9006 tumors and kidneys. The absorbed fractions for the tumor and the kidneys were assessed by Monte Carlo simulations using PENELOPE 2014 (Supplementary Materials) [45].

### SPECT/CT imaging studies

The acquisition of SPECT/CT images was performed with a dedicated small-animal SPECT/CT scanner (NanoSPECT/CT™, Mediso Medical Imaging Systems, Budapest, Hungary) as previously reported (Supplementary Material) [23, 25]. CT scans of 7.5 min duration time were followed by a SPECT scan of ~ 40 min of NF9006 tumor-bearing mice at 4 h and 24 h after injection of [^177^Lu]Lu-DOTA-folate (25 MBq, 0.5 nmol, 100 μL). During the scans, mice were anesthetized with a mixture of isoflurane and oxygen. Images were prepared using VivoQuant post-processed software (version 3.5, inviCRO Imaging Services and Software, Boston USA). A Gauss post-reconstruction filter (FWHM = 1 mm) was applied twice, and the scale of activity was indicated on the images.

### Therapy study

The subtherapeutic quantity of [^177^Lu]Lu-DOTA-folate, which was believed to sensitize tumors to ICIs, was assessed in a separate pre-therapeutic study (Supplementary Material). The design of the therapy study was adapted from Demaria et al. [15]. The experiment was performed with four groups of NF9006 tumor-bearing mice (*n* = 11) with an average initial tumor volume of 70–110 mm^3^ and an initial body weight of ~21 g (Table 1). The [^177^Lu]Lu-DOTA-folate (5 MBq; 0.5 nmol) was diluted in PBS (100 μL) containing 0.05% BSA (vehicle) and injected into a lateral tail vein. The immunoglobulin G (polyclonal Syrian hamster IgG, InVivoMab, BioXCell; 200 μg) or anti-mouse CTLA-4 monoclonal antibody (anti-CTLA-4 antibody, InVivoMab, clone 9H10, BioXCell; 200 μg) were applied intraperitoneally in 200 μL dilution buffer. Control mice (group A) were sham-treated with vehicle on day 0 and a control antibody (IgG) at days 1, 4, and 7. Mice of group B received [^177^Lu]Lu-DOTA-folate and IgG, and mice of group C received the vehicle and anti-CTLA-4 antibody in an analogous sequence. Mice of group D received [^177^Lu]Lu-DOTA-folate and anti-CTLA-4 antibody (Table 1). The mice were monitored by general observation and measuring the tumor size and body weight (Supplementary Material). Endpoint criteria, which required euthanasia of the mice, were defined as (i) a tumor volume of ≥ 1000 mm^3^, (ii) body weight loss of ≥ 15%, (iii) a combination of a tumor size of ≥ 800 mm^3^ and body weight loss of ≥ 10% and/or (iv) ulceration of the tumor and/or (v) abnormal behavior, indicating pain or unease.

**Table 1:** Design of the therapy study and tumor volumes and body weights at therapy start

Group (*n* = 11)	Treatment		Tumor volume^1^ (mm^3^) (average ± SD)	Body weight^1^ (g) (average ± SD)
	[^177^Lu]Lu-DOTA-folate (applied at day 0)	Antibody (applied at days 1, 4, and 7)	Day 0	Day 0
A	Vehicle	Control IgG antibody (200 μg/day)	110 ± 51	22.3 ± 1.8
B	[^177^Lu]Lu-DOTA-folate (5 MBq)	Control IgG antibody (200 μg/day)	71 ± 44^2^	21.3 ± 1.6^2^
C	Vehicle	anti-CTLA-4 antibody (200 μg/day)	98 ± 75^2^	21.2 ± 1.6^2^
D	[^177^Lu]Lu-DOTA-folate (5 MBq)	anti-CTLA-4 antibody (200 μg/day)	88 ± 67	21.4 ± 1.6

^*^Vehicle: 0.05% BSA in PBS

^1^No significant differences determined between the values measured for each group (*p* > 0.05)

^2^ These values refer to the *n* = 10 mice after exclusion of one mouse in group B and C, respectively

### Assessment of the therapy

The tumor growth inhibition (*TGI)* was defined as [100 − (*RTV*_T_ / *RTV*_C_ × 100)] where *RTV*_T_ is the relative tumor volume of treated mice at day 8, when the first mouse of the control group (group A) reached the endpoint, and *RTV*_C_ is the average relative tumor volume of control mice. The *TGD*_2_, *TGD*_5_ and *TGD*_8_ were calculated as the time required for the tumor volume to increase 2-, 5-fold and 8-fold, respectively, over the initial volume at the day 0. The tumor growth delay index (*TGDI*) was calculated as the *TGD*_2_, *TGD*_5_ and *TGD*_8_ ratio of treated mice (*T*) over control mice (*C*) [*TGDI*_x_ = *TGD*_x_(*T*)/*TGD*_x_(*C*); x = 2, 5 or 8].

Potential early side effects were assessed by comparison of average body weights, blood plasma chemistry, and organ mass-to-brain mass ratios (Supplementary Material).

### Statistical analysis

Statistical analysis of FR-specific binding of [^177^Lu]Lu-DOTA-folate determined in cell uptake and autoradiography studies was performed using one-way ANOVA with Bonferroni’s multiple comparisons post-test. Biodistribution data were analyzed using two-way ANOVA with Sidak’s multiple comparisons post-test. The initial body weight and tumor volume of therapy mice were tested for statistical significance using one-way ANOVA with Dunnett’s multiple comparisons post-test. Survival of mice was analyzed with Kaplan-Meier curves and a log-rank test (Mantel-Cox). All analyses were performed using GraphPad Prism (version 7.0). A *p* value of < 0.05 was considered statistically significant.

## Results

### In vitro characterization of the NF9006 breast cancer cell line for FR-targeting

Western blot analysis demonstrated FR expression in NF9006 tumor cells at ~ 30-fold lower levels than in KB tumor cells, which are known to express the FR at non-physiologically high levels (Supplementary Material Fig. S2) [42].

**Fig. 1:**
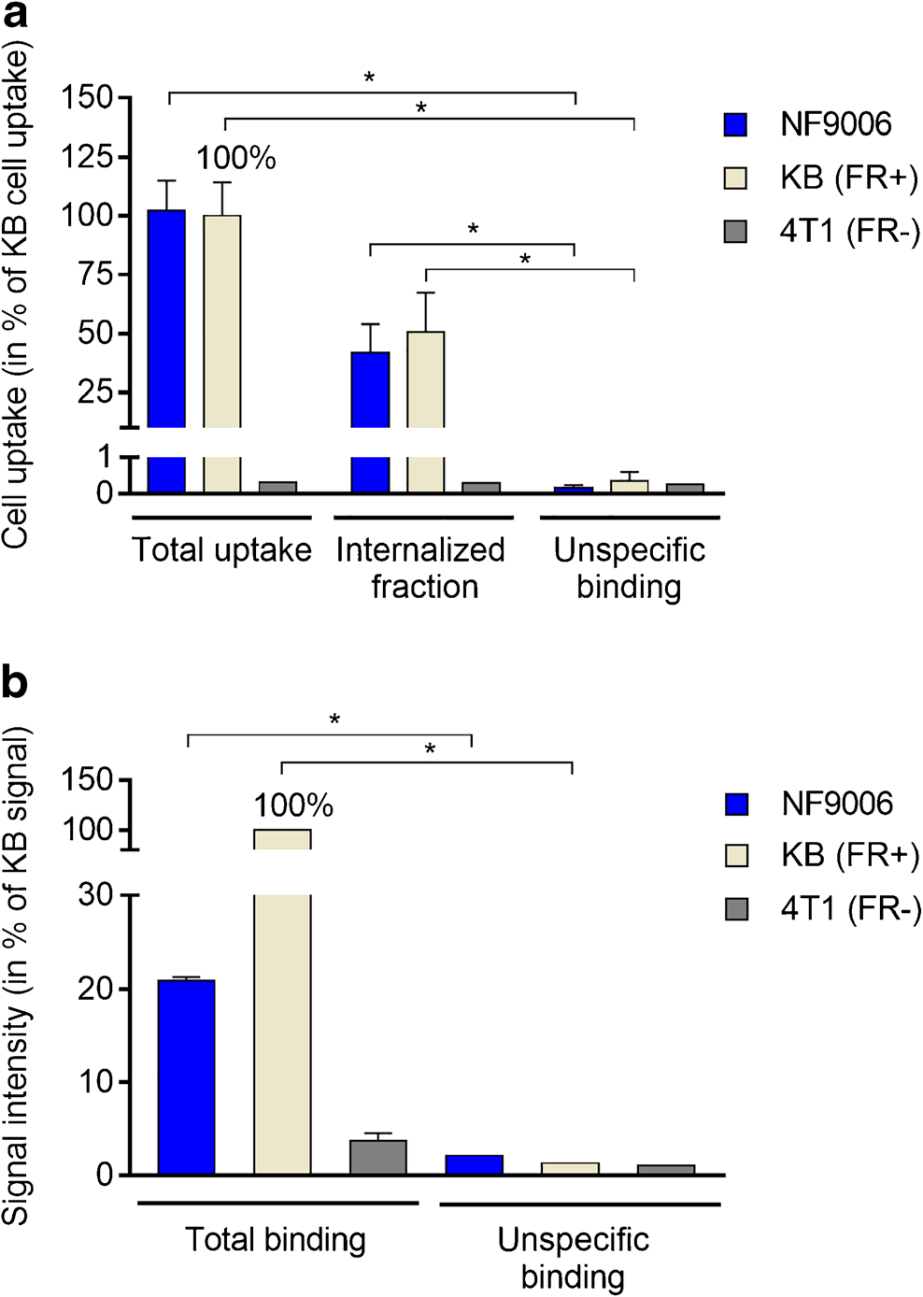
**a** Cell uptake and internalization of [^177^Lu]Lu-DOTA-folate into NF9006, KB (FR-positive), and 4T1 (FR-negative) tumor cells including blocking experiments performed with excess folic acid (average ± SD, *n* = 3–4). **b** Quantification of the autoradiographic signals obtained upon binding of [^177^Lu]Lu-DOTA-folate to NF9006, KB, and 4T1 tumors sections in the absence and presence of folic acid (average ± SD, *n* = 2). The results are presented relative to the signal intensity of KB sections (set as 100%)

Cell uptake and internalization studies revealed similar uptake of [^177^Lu]Lu-DOTA-folate into NF9006 cells (102 ± 13%) as found for KB cells (set as 100%) after a 4-h incubation period with about 50% of the activity internalized. Co-incubation with excess folic acid reduced the uptake of [^177^Lu]Lu-DOTA-folate to < 1% (*p* < 0.05), which corresponded to the uptake in FR-negative 4T1 cells (~ 0.3%) (Fig. 1a). Experiments to determine the FR-binding affinity of [^177^Lu]Lu-DOTA-folate in NF9006 tumor cells revealed a K_D_ value of 2.1 ± 0.8 nM, but the *B*_max_ value determined for NF9006 tumor cells was ~ 35-fold lower than in KB tumor cells (Supplementary Material Fig. S3).

In vitro autoradiographic images confirmed FR expression on NF9006 tumor sections, which—according to the signal intensity—was about 5-fold lower (21 ± 1%) than the signal in KB tumors (set as 100%). The binding dropped to only ~ 2% (*p* < 0.05) on sections co-incubated with excess folic acid to block FRs, similarly to the signal for FR-negative 4T1 tumors (~ 4%) (Fig. 1b; Supplementary Material Fig. S4).

### Assessment of the NF9006 tumor mouse model for FR-targeting

**Fig. 2:**
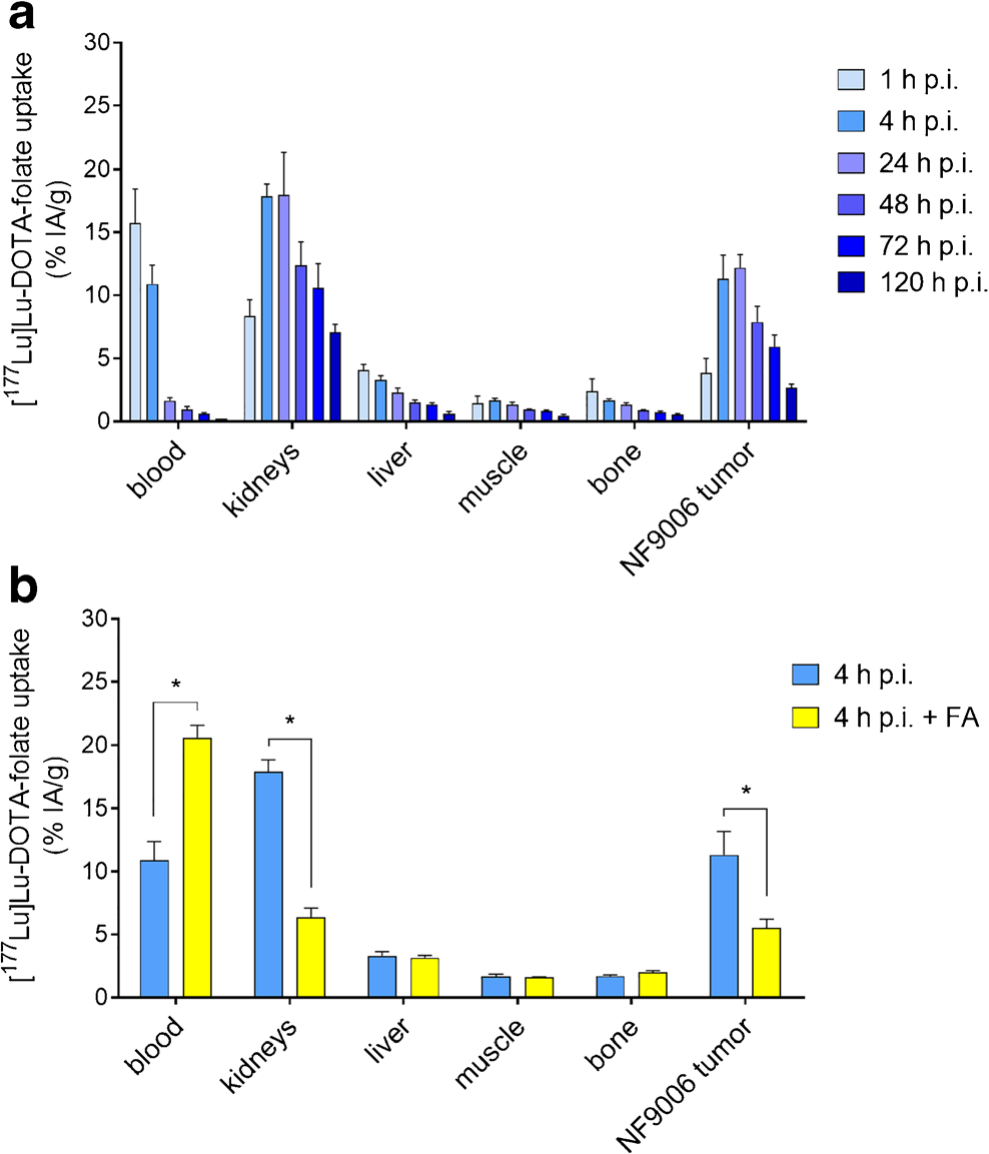
**a** Graph representing the uptake of [^177^Lu]Lu-DOTA-folate over a period of 5 days. **b** Graph representing the uptake of activity at 4 h p.i. of [^177^Lu]Lu-DOTA-folate with and without pre-injected folic acid (FA). The data are decay-corrected and expressed as percentage of injected activity per gram tissue (% IA/g), reported as average ± SD obtained from each group of mice (*n* = 3–4)

The NF9006 tumor mouse model was assessed regarding the ability to accumulate [^177^Lu]Lu-DOTA-folate in tumors (Fig. 2a, Supplementary Material Table S1). Significant uptake and retention was found in the tumor tissue with a maximum value of ~ 12% IA/g between 4 and 24 h after injection of [^177^Lu]Lu-DOTA-folate. Uptake of [^177^Lu]Lu-DOTA-folate in the kidneys (18 ± 1% IA/g; 4 h p.i.) was relatively high due to renal expression of the FR [44]. Off-target organs and tissues that do not express the FR did not substantially accumulate the [^177^Lu]Lu-DOTA-folate. Blocking studies using excess folic acid reduced the tumor and kidney uptake of [^177^Lu]Lu-DOTA-folate to ~ 50% and ~ 35%, respectively, of unblocked accumulation at 4 h after injection (Fig. 2b, Supplementary Material Table S1).

Dose estimations based on non-decay-corrected biodistribution data for NF9006 tumors and kidneys revealed a mean absorbed tumor dose of 0.7 Gy/MBq and a mean absorbed kidney dose of 1.21 Gy/MBq. The resulting tumor-to-kidney dose ratio was ~ 0.6.

### SPECT/CT imaging studies

**Fig. 3:**
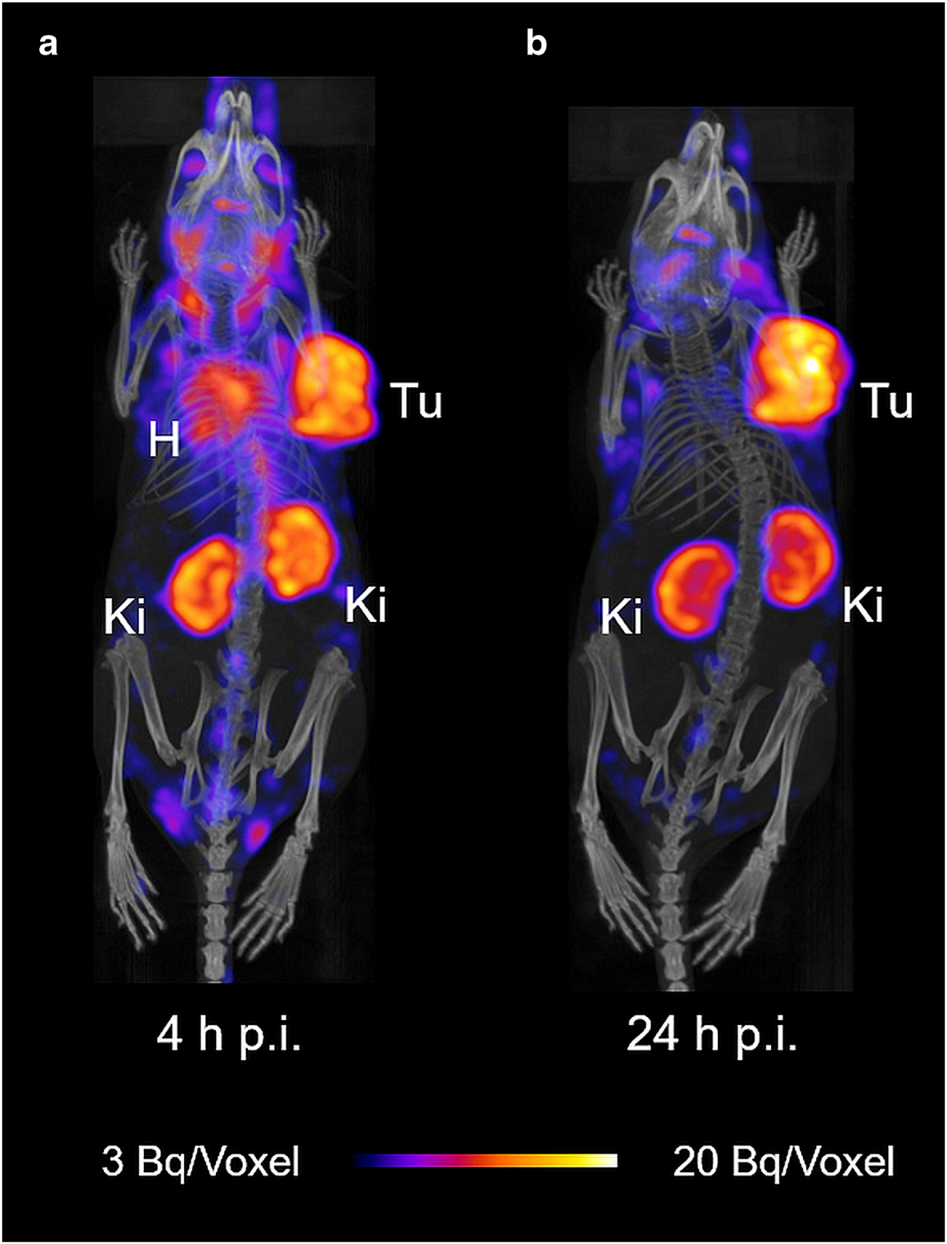
SPECT/CT images of a NF9006 tumor-bearing mouse after injection of [^177^Lu]Lu-DOTA-folate (25 MBq; 0.5 nmol per mouse) shown as maximum intensity projections (MIPs). Mouse images obtained **a** at 4 h p.i. and **b** at 24 h p.i. Accumulation of activity is visible in NF9006 tumors (Tu) and kidneys (Ki) and in the heart (H). Activity uptake was also observed in lymph nodes of the neck and armpit regions as well as in the choroid plexus

SPECT/CT imaging studies confirmed the high uptake of [^177^Lu]Lu-DOTA-folate in NF9006 tumors and in the kidneys (Fig. 3). The uptake in lymph nodes of the neck and armpits appeared specific to this mouse strain rather than related to the tumor as demonstrated in control experiments, performed with FVB mice without tumors in which the same distribution pattern was observed (Supplementary Material Fig. S5).

### Therapy study using [^177^Lu]Lu-DOTA-folate and an anti-CTLA-4 antibody

**Fig. 4:**
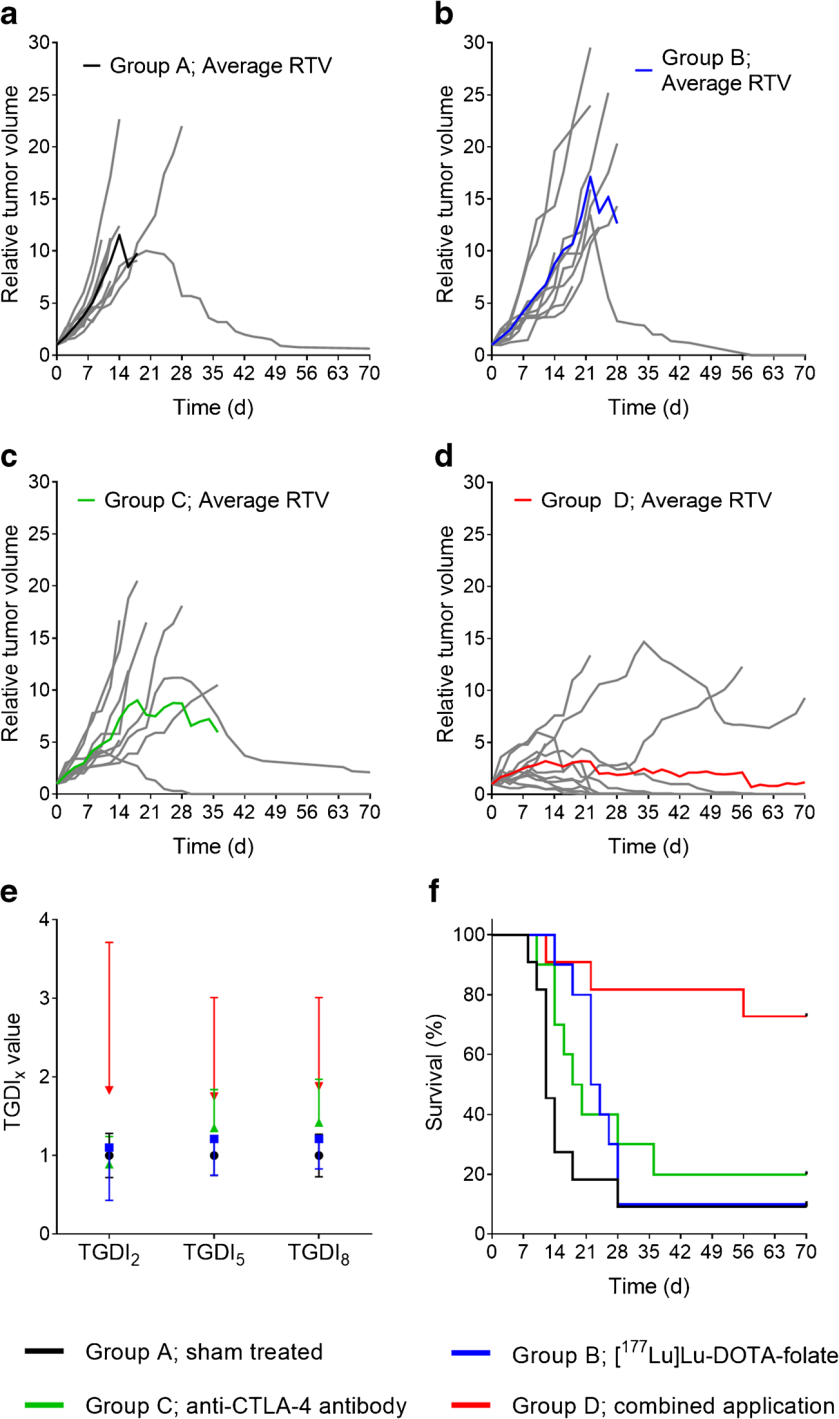
**a–d** Graphs representing the relative tumor volumes (RTV) of mice in each group. **a** RTV of mice that received PBS (group A), **b** [^177^Lu]Lu-DOTA-folate (group B), **c** anti-CTLA-4 antibody (group C), and **d** a combination of both (group D). **e** TGDI_2_, TGDI_5_, and TGDI_8_ determined for respective groups. **f** Kaplan-Meier plot of groups A–D

Based on the in vitro data and biodistribution studies, the NF9006 tumor mouse model appeared useful to investigate the potential of [^177^Lu]Lu-DOTA-folate to enhance anti-CTLA-4 immunotherapy. A subtherapeutic quantity of [^177^Lu]Lu-DOTA-folate (3.5 Gy tumor dose; 5 MBq/mouse) was chosen to obtain a low-dose radiation stimulus of the tumor prior to immunotherapy with an anti-CTLA-4 antibody (Supplementary Material Fig. S6 and Table S2). In control mice (group A), the tumors increased in size over the whole time of investigation (Fig. 4a). Mice of groups B and C, which received either [^177^Lu]Lu-DOTA-folate or the anti-CTLA-4 antibody, respectively (Fig. 4b,c), showed only ~ 10–40% delayed tumor growth compared with the controls (Fig. 4e, Supplementary Material Table S3). Mice that received [^177^Lu]Lu-DOTA-folate and anti-CTLA-4 immunotherapy (group D), responded in 8 out of 11 cases, demonstrated by decreasing tumor volumes over time, and in 7 cases the NF9006 tumors disappeared entirely (Fig. 4d). This led to increased tumor growth delay indices (TGDI) and tumor growth inhibition (TGI) of mice in group D as compared with mice of the other groups (Fig. 4e; Table 2; Supplementary Material Table S3). Mice of group D also showed the highest survival rate (~ 70% at day 70) when compared with groups A, B, and C (≤ 20%) (Fig. 4f). The median survival time of mice of group D remained undetermined since more than 50% of the mice were still alive at the end of the study. The median survival times of mice of groups B and C were 23 days and 19 days, respectively, compared with the control mice that had a median survival time of 12 days (Table 2).

**Table 2:** Comparison of euthanasia period and median survival of mice of the therapy study

Group	Treatment	Time frame of euthanasia (day)	Median survival (days)	Survival curve sig. different from groups ^1^	TGI (%)
A	Sham treated	8–70	12	D	0
B	[^177^Lu]Lu-DOTA-folate	14–70	23	D	7 ± 50
C	Anti-CTLA-4 antibody	10–70	19	D	17 ± 37
D	Combination	12–70	>70^2^	A, B, C	48 ± 28

*Day 70 = End of the study

^1^Comparison of survival curves by log-rank (Mantel-Cox) test

^2^more than 50% of mice were alive at the end of the study at day 70

All mice that were euthanized before the end of the study reached the endpoint due to the tumor volume and ulceration, but not due to body weight loss. No significant differences were observed among treated animals (groups B–D) and untreated controls (group A) regarding blood plasma parameters and organ-to-brain mass ratios at the time of euthanasia as well as in terms of body weight, which indicates the absence of early adverse effects (Supplementary Material Fig. S7 and Tables S4, S5, and S6).

## Discussion

In this study, we set out to evaluate the syngeneic NF9006 breast cancer mouse model with the aim to use it for the investigation of anti-CTLA-4-based immunotherapy after low-dose irradiation delivered by application of [^177^Lu]Lu-DOTA-folate.

The feasibility of targeting NF9006 breast cancer with [^177^Lu]Lu-DOTA-folate was successfully demonstrated in vitro and in vivo. The uptake of [^177^Lu]Lu-DOTA-folate in NF9006 tumor cells was comparable with the results obtained with KB tumor cells in spite of the lower FR expression level determined in NF9006 tumor cells. Obviously, the applied concentration of [^177^Lu]Lu-DOTA-folate was far below the quantity that would result in saturation effects of the receptor (Supplementary Material Fig. S8). SPECT/CT imaging and biodistribution studies with mice revealed a reasonable accumulation of [^177^Lu]Lu-DOTA-folate in NF9006 tumors which was about half of the uptake in KB tumor xenografts (unpublished results). The dose calculations revealed, however, that 5 MBq injected [^177^Lu]Lu-DOTA-folate per mouse was sufficient to obtain 3.5 Gy absorbed tumor dose. This quantity of injected [^177^Lu]Lu-DOTA-folate resulted in an absorbed kidney dose (~ 6 Gy) far below the safe limit of 23 Gy [24]. The tumor growth delay of mice that received [^177^Lu]Lu-DOTA-folate prior to anti-CTLA-4 antibody therapy was doubled, and, as a consequence, the survival time of mice substantially increased.

These data clearly indicate the promising potential of [^177^Lu]Lu-DOTA-folate to be used as a radiation stimulus to enhance breast tumor response to anti-CTLA-4 immunotherapy. It is hypothesized but not confirmed yet that the observed effect was based on radiation-induced changes in the tumor microenvironment. This may include CD8+ T cell infiltration and recruitment of innate immune cells as it was previously reported in preclinical studies that used radioconjugates to enhance the response to ICIs in mouse models of melanoma [19, 46], non-Hodgkin lymphoma [47], and colon cancer [18].

It is worth mentioning that the therapy data of our study resulted from mouse groups with tumors in a relatively large volume range, which is a relevant finding in view of the clinical situation, in which large inter- and intra-individual differences among the stages of tumor metastases are expected. It indicated that even larger tumors responded well (Supplementary Material Fig. S9), although it is known that the size of tumors may critically influence the infiltration of immune cells [48–51].

The set-up of future preclinical studies has to be carefully evaluated in order to provide robust data that are meaningful in view of clinical translation of the proposed concept. It is, thus, essential to investigate the underlying mechanism of the immune cell response upon application of [^177^Lu]Lu-DOTA-folate as previously reported for [^177^Lu]Lu-DOTATATE [52]. This may provide relevant information to allow a further optimization of the treatment scheme.

Spontaneous tumor mouse models would reflect the patient situation better; however, using transgenic MMTV-Neu mice, from which NF9006 cells are derived, would require effective means to determine the tumor burden before and during therapy. In this context, we investigated the option of positron emission tomography (PET) imaging using ^18^F-AzaFol, a clinically investigated PET folate radiotracer [53, 54], which unambiguously visualized NF9006 tumors in mice (Supplementary Material Table S7 and Figs. S10 and S11), while this was not the case when using [^18^F]fluorodeoxyglucose ([^18^F]FDG), the most frequently employed clinical PET radiotracer.

Certainly, the approach reported in this study is not limited to the use of [^177^Lu]Lu-DOTA-folate but may open new application fields for other tumor-targeting radioconjugates that may induce immunogenic cell death and, thus, enhance ICI therapy as previously reported by Rouanet et al. [46]. Similarly, other systemic therapies, such as radiosensitizing chemotherapeutics, and mTOR inhibitors may also profit from combination with tumor-targeting radioconjugates [55].

## Conclusion

In this study, we demonstrated the promising potential of [^177^Lu]Lu-DOTA-folate to enhance the tumor response to ICIs. In view of a future clinical translation, further preclinical studies are warranted for an in-depth understanding of the underlying immune response to radiation in this model. The approach of turning “cold” tumors “hot” by application of low-dosed radiopharmaceuticals may provide nuclear oncology with a new dimension in the context of future perspectives in cancer therapy.

## Electronic supplementary material

ESM 1 (DOCX 5.00 mb)

## Abbreviations

ANOVA: Analysis of variance
BSA: Bovine serum albumin
CD8+: Cluster of differentiation 8 positive
CT: Computed tomography
CTLA-4: Cytotoxic T lymphocyte-associated antigen 4
DOTA: 1,4,7,10-tetraazacyclododecane-1,4,7,10-tetraacetic acid
FA: Folic acid
FDG: Fluorodeoxyglucose
FR: Folate receptor
FWHM: Full width at half maximum
H: Heart
HPLC: High performance liquid chromatography
IA: Injected activity
ICI: Immune checkpoint inhibitor
IgG: Immunoglobulin G
Ki: Kidney
mAb: Monoclonal antibody
MMTV: Mouse mammary tumor virus
PET: Positron emission tomography
PBS: Phosphate-buffered saline
PVDF: Polyvinylidene difluoride
RBW: Relative body weight
RPMI: Roswell Park Memorial Institute
RTV: Relative tumor volume
SD: Standard deviation
SPECT: Single photon emission computed tomography
TGDI: Tumor growth delay index
TGI: Tumor growth inhibition
Tu: Tumor
