# Supplementary material for: Promising potential of [177Lu]Lu-DOTA-folate to enhance tumor response to immunotherapy—a preclinical study using a syngeneic breast cancer model
Source: Eur J Nucl Med Mol Imaging. 2020 Oct 19;48(4):984–94. doi: 10.1007/s00259-020-05054-9 (PMC8041666; doi:10.1007/s00259-020-05054-9)
Supplement: Supplementary file 1 — (DOCX 5.00 mb) [file 259_2020_5054_MOESM1_ESM.docx]

**SUPPLEMENTARY MATERIAL**

**Promising potential of [^177^Lu]Lu-DOTA-folate to enhance tumor response to immunotherapy – a preclinical study using a syngeneic breast cancer model**

Patrycja Guzik^1^, Klaudia Siwowska^1^, Hsin-Yu Fang^1^, Susan Cohrs^1^, Peter Bernhardt^2,3^, Roger Schibli^1,4^, Cristina Müller^1,4*^

1. Center for Radiopharmaceutical Sciences ETH-PSI-USZ, Paul Scherrer Institute, 5232 Villigen-PSI, Switzerland

2. Department of Radiation Physics, The Sahlgrenska Academy, University of Gothenburg, 413 45 Gothenburg, Sweden

3. Department of Medical Physics and Medical Bioengineering, Sahlgrenska University Hospital, 413 45 Gothenburg, Sweden

4. Department of Chemistry and Applied Biosciences, ETH Zurich, 8093 Zurich, Switzerland

**E-Mail addresses**:

patrycja.guzik@psi.ch; [klaudiasiwowska@gmail.com](mailto:klaudiasiwowska@gmail.com); [hy.fang11@gmail.com](mailto:hy.fang11@gmail.com); [susan.cohrs@psi.ch](mailto:susan.cohrs@psi.ch); [peter.bernhardt@gu.se](mailto:peter.bernhardt@gu.se); [roger.schibli@psi.ch](mailto:roger.schibli@psi.ch); [cristina.mueller@psi.ch](mailto:cristina.mueller@psi.ch)

***Correspondence to**:

PD Dr. Cristina Müller

Center for Radiopharmaceutical Sciences ETH-PSI-USZ

Paul Scherrer Institute

5232 Villigen-PSI

Switzerland

e-mail: cristina.mueller@psi.ch

phone: +41 56 310 44 54; fax: +41 56 310 28 49

**1. Radiolabeling of the folate conjugate**

***Purpose:*** Radiolabeling of the DOTA-folate conjugate with lutetium-177 was performed directly before application for in vitro and in vivo experiments.

***Methods:*** The DOTA-folate was labeled with lutetium-177 (no-carrier-added, in 0.05 M HCl; Medical Isotopes ITM GmbH, Germany) in a 1:5 (*v/v*) mixture of sodium acetate (0.5 M) and HCl (0.05 M) at pH ~4.5 as previously reported [1, 2]. The molar activity was commonly 50 MBq/nmol if not otherwise stated in the article. The reaction mixture was incubated for 10 min at 95 °C, followed by a quality control using HPLC. A Merck Hitachi LaChrom HPLC system, equipped with a D-7000 interface, a L-7200 autosampler, a radiation detector (LB 506 B; Berthold) and a L-7100 pump was connected with a reversed-phase C18 column (Xterra^TM^ MS, C18, 5 μm, 150 × 4.6 mm; Waters). The mobile phase consisted of Milli-Q water containing 0.1% TFA (A) and acetonitrile (B). A linear gradient of solution A (95–20%) and solvent B (5–80%) over 15 min was used at a flow rate of 1 mL/min followed by 5 min equilibration of the system. A sample of the reaction mixture was diluted in Milli-Q water containing sodium diethylenetriamine pentaacetic acid (Na-DTPA, 50 µM) for quality control using HPLC.

***Results:*** The HPLC chromatogram obtained for quality control showed high radiochemical purity (≥98%) of the [^177^Lu]Lu-DOTA-folate labeled at a molar activity of 50 MBq/nmol (Fig. S1). [^177^Lu]Lu-DOTA-folate was used for in vitro and in vivo experiments without further purification.


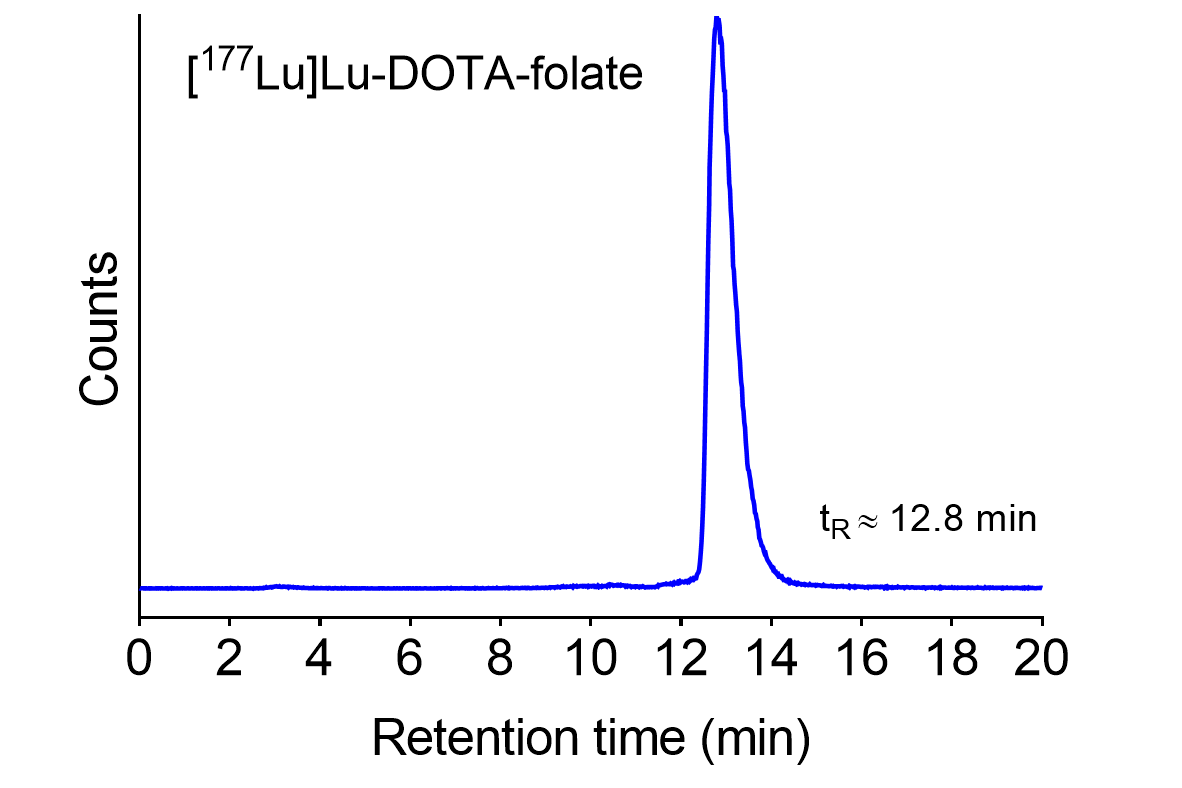


## Fig. S1 HPLC chromatogram of the DOTA-folate labeled with lutetium-177. The [^177^Lu]Lu-DOTA-folate was eluted with a retention time of t_R_ ≈ 12.8 min. The presence of unreacted lutetium-177 detected as [^177^Lu]Lu-DTPA would appear with a retention time of t_R_ ≈ 3 min.

**2. Western blot analysis**

***Purpose:*** Western blot analysis was performed to demonstrate FR expression on NF9006 tumor cells and to estimate the level of FR expression relative to the expression in KB tumor cells.

***Methods***: Cells were seeded into 6-well plates (3 × 10^6^ cells/well) in FFRPMI with supplements and incubated overnight at 37 °C and 5% CO_2_ to form a monolayer. Cell lysates were prepared using 50–70 µL cell lysis buffer (RIPA lysis and extraction buffer, 89900, Thermo Scientific), with a protease inhibitor cocktail (cOmplete^TM^, Roche). Lysates containing 60 µg protein of NF9006 and 4T1 cells or 10 µg protein of KB cells were mixed with loading buffer containing dithiothreitol (DTT) and separated by sodium dodecyl sulfate polyacrylamide gel electrophoresis (SDS-PAGE). Proteins were transferred to a polyvinylidene fluoride membrane. Skim milk (5%) in Tris-buffered saline containing 0.05% Tween^TM^ (TBST, pH 7.5) was used to prevent unspecific binding of the antibody. Incubation with the primary anti-FR antibody (Abcam, rabbit antibody, ab67422, 1:1800) was performed overnight at 4 °C. A secondary anti-rabbit IgG antibody (Cell Signaling, goat antibody, 7074S, 1:3000) functionalized with horseradish peroxidase was used together with Amersham ECL (enhanced chemiluminescence) Prime Western Blotting Detection Reagent (GE Healthcare) for signal detection. Detection of GAPDH served as a protein loading control (Cell Signaling, 5174S, rabbit mAb, 1:2000 and (HRP)-conjugated anti-rabbit IgG, 7074S, 1:5000). The results were quantified based on the peaks in the profile plot obtained in ImageJ software (version 1.52d). The intensity of the bands of the FR were standardized to the respective signal of GAPDH band, and put into relation to the signal obtained with KB cells (set as 100%). The results were obtained from several western blots.

***Results:*** The results are reported in the main article and shown in Fig. S2.

**
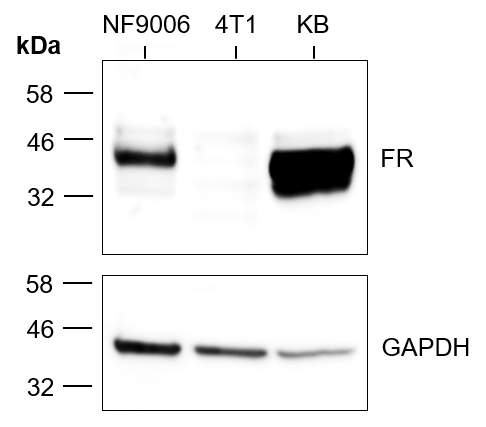
**

**Fig. S2. Image of a representative western blot.** The signal of NF9006 tumor cells (60 μg) is shown in comparison to the signals of 4T1 tumor cells (60 μg) as a negative control and KB tumor cells
(10 μg) as a FR-positive control. GAPDH staining was performed as a loading control.

**3. Cell uptake and internalization**

***Purpose:*** Uptake and internalization studies of [^177^Lu]Lu-DOTA-folate were performed to demonstrate FR-specific accumulation in NF9006 tumor cells and to compare the results with those obtained with KB and 4T1 tumor cells, respectively.

***Methods:*** Uptake and internalization of [^177^Lu]Lu-DOTA-folate (25 MBq/nmol) was determined as previously reported [2]. NF9006 tumor cells were seeded in 12-well plates (~7×10^5^ cells in 2 mL FFRPMI culture medium/well) and incubated at 37 °C and 5% CO_2_ to allow adhesion and growth overnight. The cells were washed with PBS prior to the addition of FFRPMI cell culture medium (975 μL/well) and [^177^Lu]Lu-DOTA-folate (37.5 kBq, 25 μL, 1.5 pmol per well). The well-plates were incubated for 4 h at 37 °C and 5% CO_2_. To determine the uptake of [^177^Lu]Lu-DOTA-folate, tumor cells were washed with ice-cold PBS. The internalized fraction was determined in cells washed with stripping buffer (aqueous solution of 0.1 M acetic acid and 0.15 M NaCl, pH 3). All cell samples were lysed by addition of NaOH (1 M, 1 mL) to each well and measured in a γ-counter (Perkin Elmer, Wallac Wizard 1480). The protein concentration was determined for each sample using a Micro BCA Protein Assay kit (Pierce, Thermo Scientific) in order to standardize the measured activity (percentage of total added activity) to the amount of proteins in each well. The experiments were performed at least three times in triplicate. Cell internalization experiments with KB and 4T1 tumor cells were performed in analogy, however, normal RPMI medium was used for 4T1 tumor cell experiments.

***Results:*** The results of uptake and internalization of [^177^Lu]Lu-DOTA-folate into NF9006 tumor cells and control cells (KB and 4T1 tumor cells) are reported in the main article (Fig. 1a).

**4. Determination of the K_D_ value of [^177^Lu]Lu-DOTA-folate using NF9006 tumor cells**

***Purpose*:** FR-binding affinity of [^177^Lu]Lu-DOTA-folate (K_D_ value) was determined using NF9006 cells.

***Methods:*** The FR-binding affinity of [^177^Lu]Lu-DOTA-folate was determined as previously reported using NF9006 tumor cells [2]. NF9006 tumor cells were seeded in 48-well plates (~0.25 × 10^6^ cells in 0.5 mL FFRPMI with supplements), and incubated at 37 °C and 5% CO_2_ to allow cell attachment overnight. The experiments were performed on ice using ice-cold buffers and reagents. The NF9006 tumor cells were washed with PBS pH 7.4 before adding fresh FFRPMI without additives (450 μL per well) and diverse dilutions of [^177^Lu]Lu-DOTA-folate (20 MBq/nmol; 50 μL) to obtain final folate concentrations of 0.1 nM–500 nM per well. Unspecific binding of [^177^Lu]Lu-DOTA-folate was determined in separate cell samples co-incubated with excess folic acid (100 μM) to block the FRs. After incubation of the cells for 1 h at 4 °C, and several washing steps, the cells were lysed using a sodium hydroxide solution (1 M, 0.5 mL per well). The cell suspensions were transferred into 3.5 mL-tubes for measurement in a γ-counter (Perkin Elmer Wallac Wizard 1480). The reported values are the average ± SD obtained from three independent experiments performed with three replicates. The data were fitted with a one-site binding saturation curve using GraphPad Prism software (version 7) in order to determine the K_D_ value of [^177^Lu]Lu-DOTA-folate.

***Results:*** The K_D_ value of [^177^Lu]Lu-DOTA-folate was determined as 2.1 ± 0.8 nM which is in the same range as previously determined for albumin-binding folate radioconjugates using IGROV-1 tumor cells (Fig. S3) [2].


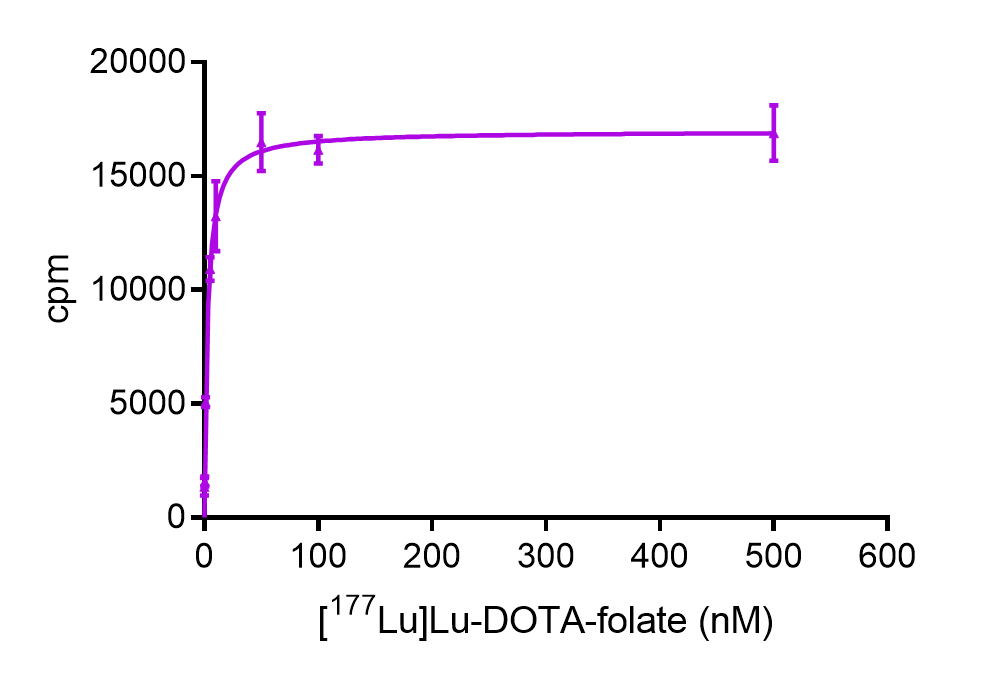


**Fig. S3.** Representative [^177^Lu]Lu-DOTA-folate binding curve performed with NF9006 tumor cells to determine the K_D_ value.

**5. Determination of FR expression in NF9006 cells relative to KB cells**

***Purpose*:** In order to compare the level of FR expression (B_max_) between NF9006 and KB cells, saturation experiments were performed using [^177^Lu]Lu-DOTA-folate.

***Methods:*** NF9006 and KB tumor cells, respectively, were seeded in 48-well plates (~0.25 × 10^6^ cells in 0.5 mL FFRPMI with supplements), and incubated at 37 °C and 5% CO_2_ to allow cell attachment overnight. [^177^Lu]Lu-DOTA-folate (20 MBq/nmol) was diluted in PBS to obtain folate concentrations up to 5 μM and 20 μM for NF9006 and KB tumor cells, respectively. The experiment was performed on ice in order to prevent FR internalization. The tumor cells were washed with ice-cold PBS pH 7.4 before adding fresh FFRPMI without additives (450 μL per well) and [^177^Lu]Lu-DOTA-folate (50 μL) to obtain final folate concentrations in the range of up to 500 nM and up to 2 μM per well for NF9006 and KB cells, respectively. Unspecific binding of [^177^Lu]Lu-DOTA-folate was determined in separate cell samples which were co-incubated with excess folic acid (100 μM) to block surface-exposed FRs. After incubation of the cells by shaking the well plates for 1 h at 4 °C, cells were washed twice with PBS to remove unbound [^177^Lu]Lu-DOTA-folate followed by cell lysis using sodium hydroxide solution (1 M, 0.5 mL). The cell suspensions were transferred into 3.5 mL-tubes for measurement in a γ-counter (Perkin Elmer Wallac Wizard 1480). The data were fitted with a one-site binding saturation curve using GraphPad Prism software (version 7) in order to determine the plateau and reading the counts of the B_max_ value.

***Results:*** Determination of B_max_ levels revealed 30­- to 40-fold higher counts for KB cells than for NF9006 tumor cells. These findings clearly indicated a substantially lower FR expression level in NF9006 tumor cells as compared to the expression level in KB cells, which are known to express the FR at unnaturally high levels.

**6. In vitro autoradiography**

***Purpose:*** In vitro autoradiography was performed in order to test the binding of [^177^Lu]Lu-DOTA-folate on frozen sections of NF9006 tumors and, therewith confirm FR expression of NF9006 tumors. The results were compared with those obtained with FR-positive KB tumor and FR-negative 4T1 tumor sections, respectively.

***Methods:*** In vitro autoradiography studies were performed according to a previously published procedure [3]. NF9006 tumors were grown in FVB mice, KB tumors were grown in CD-1 athymic nude mice and 4T1 tumors were grown in normal BALB/c mice. The collected tumors were embedded in TissueTek (Cryo-M-Bed, Bright) and frozen at –80 °C. Tumor tissue sections of 5–10 µm thickness were prepared using a cryotome (Bright OTF Cryostat, OTF/AS-001/MR/V/304/X, Huntingdon, England). Slides were thawed and incubated in Tris-buffer (167 mM Tris-HCl, 5 mM MgCl_2_) containing 0.25% BSA for 10 min at room temperature (RT). [^177^Lu]Lu-DOTA-folate (50 MBq/nmol) was diluted in Tris-buffer containing 1% BSA (0.5 MBq/mL; corresponding to 0.01 µM folate) and added on the tissue sections (100 µL). Excess folic acid (100 µM) was used to block FRs. After incubation of the sections for 1 h at RT, the slides were washed several times and air-dried. Autoradiographic images were obtained using a storage phosphor system (Cyclone Plus, Perkin Elmer) and the signal intensity was quantified using OptiQuant software (version 5.0, Bright Instrument Co Ltd, Perkin Elmer). The signals obtained from NF9006 and 4T1 tumor sections were expressed relative to the signal obtained for KB tumor sections (set as 100%). Representative images were prepared using Adobe Photoshop CC (version 2017).

***Results:*** The results are reported in the main article (Fig. 1b). Representative autoradiographic images are shown in Fig. S4.

**
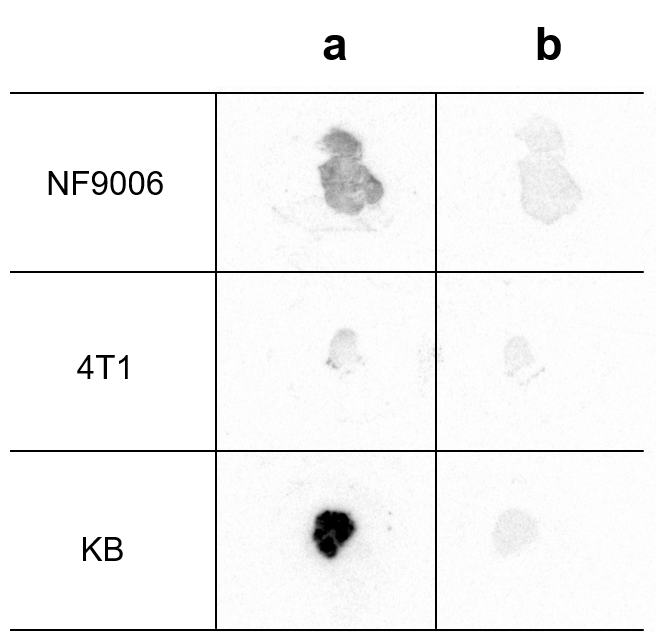
**

## Fig. S4 Representative images of in vitro autoradiographic images obtained after exposure of NF9006, 4T1 and KB tumor sections to [^177^Lu]Lu-DOTA-folate. (a) Panel showing total binding of [^177^Lu]Lu-DOTA-folate obtained by incubating tumor sections with [^177^Lu]Lu-DOTA-folate. (b) Panel showing unspecific binding upon incubation of tumor sections with [^177^Lu]Lu-DOTA-folate and excess folic acid to block FRs.

**7. Biodistribution studies**

***Purpose:*** Biodistribution studies of [^177^Lu]Lu-DOTA-folate were performed in NF9006 tumor-bearing mice to confirm the feasibility of FR targeting and for quantification of accumulated [^177^Lu]Lu-DOTA-folate in the tumor tissue and kidneys for dose estimations.

***Methods:*** The methods of the biodistribution studies are reported in the main article.

***Results:*** The data were decay-corrected and listed as percentage of the injected activity per gram of tissue mass (% IA/g) (Table S1). A graph representing the values for the most important organs and tissues is shown in the main article (Fig. 2).

**Table S1** Biodistribution data of [^177^Lu]Lu-DOTA-folate in NF9006 tumor-bearing mice obtained at variable timepoints post injection (p.i.). The data are decay-corrected and listed as percentage of injected activity per gram tissue (% IA/g), reported as average ± SD obtained from each group of mice (n=3–4)

|  | **[^177^Lu]Lu-DOTA-folate** | | | | | | |  |
| --- | --- | --- | --- | --- | --- | --- | --- | --- |
|  | **1 h p.i.** | **4 h p.i.** | **24 h p.i.** | **2 days p.i.** | **3 days p.i.** | **5 days p.i.** | **4 h p.i. + folic acid^1)^** | |
| Blood | 16 ± 3 | 11 ± 2 | 1.7 ± 0.3 | 1.0 ± 0.2 | 0.60 ± 0.10 | 0.21 ± 0.00 | 21 ± 1 | |
| Heart | 6.0 ± 1.3 | 5.6 ± 0.8 | 4.1 ± 0.2 | 2.8 ± 0.2 | 2.6 ± 0.2 | 1.3 ± 0.1 | 7.4 ± 0.6 | |
| Lung | 6.7 ± 1.1 | 6.4 ± 0.6 | 2.6 ± 0.4 | 1.7 ± 0.1 | 1.5 ± 0.2 | 0.76 ± 0.18 | 9.0 ± 0.8 | |
| Spleen | 2.0 ± 0.1 | 1.6 ± 0.1 | 0.80 ± 0.14 | 0.63 ± 0.09 | 0.55 ± 0.05 | 0.36 ± 0.02 | 2.4 ± 0.1 | |
| Kidneys | 8.3 ± 1.3 | 18 ± 1 | 18 ± 3 | 12 ± 2 | 11 ± 2 | 7.1 ± 0.7 | 6.4 ± 0.7 | |
| Stomach | 2.7 ± 1.2 | 1.7 ± 0.3 | 0.78 ± 0.31 | 0.72 ± 0.07 | 0.57 ± 0.15 | 0.38 ± 0.05 | 1.9 ± 0.3 | |
| Intestines | 1.4 ± 0.2 | 1.3 ± 0.3 | 0.39 ± 0.03 | 0.32 ± 0.12 | 0.25 ± 0.04 | 0.13 ± 0.02 | 2.0 ± 0.5 | |
| Liver | 4.1 ± 0.5 | 3.3 ± 0.3 | 2.3 ± 0.4 | 1.5 ± 0.2 | 1.3 ± 0.1 | 0.63 ± 0.19 | 3.2 ± 0.2 | |
| Salivary gl. | 6.2 ± 0.4 | 6.4 ± 0.5 | 5.1 ± 0.6 | 3.8 ± 0.3 | 3.4 ± 0.5 | 2.7 ± 0.3 | 3.3 ± 0.3 | |
| Muscle | 1.4 ± 0.6 | 1.7 ± 0.2 | 1.3 ± 0.2 | 0.93 ± 0.08 | 0.82 ± 0.11 | 0.46 ± 0.12 | 1.6 ± 0.1 | |
| Bone | 2.4 ± 1.0 | 1.7 ± 0.1 | 1.3 ± 0.2 | 0.90 ± 0.03 | 0.74 ± 0.10 | 0.54 ± 0.09 | 2.0 ± 0.2 | |
| NF9006 Tumor | 3.9 ± 1.2 | 11 ± 2 | 12 ± 1 | 7.9 ± 1.3 | 5.9 ± 0.9 | 2.7 ± 0.3 | 5.5 ± 0.7 | |
| Tu-to-blood | 0.25 ± 0.09 | 1.1 ± 0.3 | 7.6 ± 1.9 | 8.7 ± 3.7 | 10 ± 4 | 13 ± 1 | 4.7 ± 5.3 | |
| Tu-to-liver | 0.97 ± 0.37 | 3.4 ± 0.5 | 5.4 ± 1.1 | 5.3 ± 1.3 | 4.5 ± 1.0 | 4.5 ± 1.0 | 2.3 ± 2.4 | |
| Tu-to-kidney | 0.46 ± 0.07 | 0.63 ± 0.10 | 0.69 ± 0.11 | 0.64 ± 0.07 | 0.56 ± 0.04 | 0.38 ± 0.06 | 0.31 ± 0.37 | |

^1)^ excess folic acid (100 μg) was injected immediately before [^177^Lu]Lu-DOTA-folate to block FRs.

**8. Dosimetric calculations**

***Purpose:*** Dosimetric calculations were performed to estimate the absorbed dose to tumors and kidneys after administration of the [^177^Lu]Lu-DOTA-folate.

***Methods:*** Bi-exponential functions were used to describe the tissue biokinetics of [^177^Lu]Lu-DOTA-folate. Uncertainties in the time integrated activity concentration coefficients (TIACCs) were generated by using multiple unique biokinetic curves for tumor xenografts and kidneys. These curves were generated by selecting one of the measured tissue activity concentrations (MTACs) per timepoint only. Thereby, several unique biokinetic curves were generated by selecting different MTACs per timepoint for each curve. For each biokinetic curve a bi-exponential curve fit was performed using the software MATLAB (MathWorks, Torrance, California, USA). The TIACCs were obtained by integrating the generated bi-exponential functions to infinity. The mean and standard deviation of TIACC was calculated for [^177^Lu]Lu-DOTA-folate. The specific mean absorbed dose *D* for the NF9006 tumor and kidneys was calculated by:

$D=TIACC\cdot\left( \sum_{i} {E_{i}\gamma_{i}\emptyset}_{i} \right)\cdot\alpha$ (1)

where *E_i_* is the energy emitted of the *i*^th^ radiation with a frequency per decay of *γ*_i_; *∅_i_* the absorbed energy fraction within an organ and *α =* 5.76 ‧ 10^-7^ the conversion factor to convert to the unit Gy/MBq. The absorbed fractions were calculated by Monte Carlo simulation using PENELOPE 2014 [4]. In the simulations, spherical shapes of the organs were assumed. The decay data of lutetium-177 were obtained from ICRU 107 (www.nucleide.org).

***Results:*** The results are reported in the main article.

**9. SPECT/CT imaging studies**

***Purpose:*** SPECT/CT experiments were performed with NF9006 tumor-bearing mice to visualize the accumulation of [^177^Lu]Lu-DOTA-folate in vivo. Additional SPECT/CT scans of non-tumor-bearing mice were performed to compare the accumulation of activity in lymph nodes and determine on whether it was related to the tumors.

***Methods:*** The acquisition of SPECT/CT images was performed with a dedicated small-animal SPECT/CT scanner (NanoSPECT/CT^TM^, Mediso Medical Imaging Systems, Budapest, Hungary) as previously reported [34, 35]. CT scans of 7.5 min duration time were followed by a SPECT scan of ~40 min at 4 h and 24 h after intravenous injection of the mice with [^177^Lu]Lu-DOTA-folate (25 MBq, 0.5 nmol, 100 μL PBS pH 7.4 with 0.05% BSA). During the scans, mice were anesthetized with a mixture of isoflurane and oxygen. The images were acquired using Nucline Software (version 1.02, Mediso Ltd., Budapest, Hungary). The reconstruction of SPECT data was performed using HiSPECT software (version 1.4.3049, Scivis GmbH, Göttingen, Germany). Images were prepared using VivoQuant post-processed software (version 3.5, inviCRO Imaging Services and Software, Boston USA). A Gauss post-reconstruction filter (FWHM = 1 mm) was applied twice and the scale of activity was set as indicated on the images (minimum value = 3 Bq/voxel to maximum value = 20 Bq/voxel). The activity in the mice was measured immediately after injection of [^177^Lu]Lu-DOTA-folate (set as 100%) and before scanning to allow determination of the percentage activity retained in the mice based on these non-decay corrected activity measurements.

***Results:*** The SPECT/CT images and results are described in the main article and shown in Fig. 3. The amount of [^177^Lu]Lu-DOTA-folate injected into the mouse bearing a NF9006 tumor was 27 MBq [^177^Lu]Lu-DOTA-folate (Fig. 3a/b). In this case, ~93% of the injected activity (25 MBq) was measured in the mouse body after 4 h and ~67% (18 MBq) after 24 h p.i.

SPECT/CT images with NF9006 tumor bearing mice showed distinct accumulation of activity in the lymph nodes at the neck and armpits (Fig. 3), which was in contradiction to the observations made with commonly used athymic nude mice [2]. Additional SPECT/CT images of FVB/NCrl mice without tumors confirmed accumulation of activity in the lymph nodes, indicating that it was unrelated to the tumor (Fig. S5).


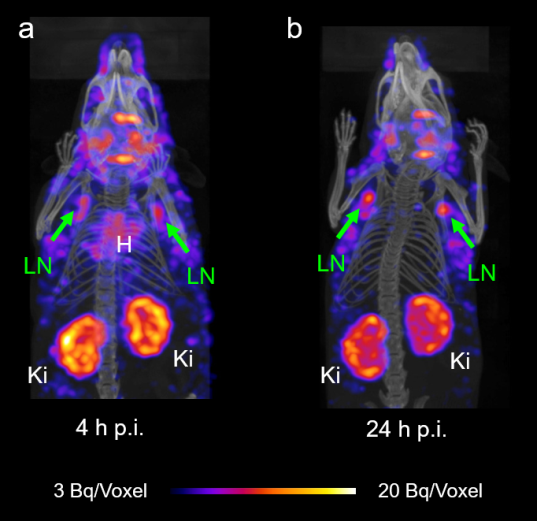


## Fig. S5 Additional SPECT/CT images of accumulated activity in the lymph nodes of FVB mice without tumors. (a) SPECT/CT scan acquired 4 h after injection of [^177^Lu]Lu-DOTA-folate; (b) SPECT/CT scan acquired 24 h after injection of [^177^Lu]Lu-DOTA-folate. LN = lymph node; Ki = kidney; H = heart.

**10. NF9006 tumor response to [^177^Lu]Lu-DOTA-folate administration**

***Purpose:*** [^177^Lu]Lu-DOTA-folate was applied to NF9006 tumor-bearing mice to determine the maximum absorbed tumor dose of [^177^Lu]Lu-DOTA-folate, which did not have a substantial effect on the tumor growth.

***Methods:*** The methods employed for this study were identical to those applied for the immunotherapy study reported in the main article. In brief, mice with NF9006 tumors were divided in three groups (n=5) and injected with vehicle (PBS containing 0.05% BSA) or 5 MBq and 10 MBq [^177^Lu]Lu-DOTA-folate to deliver a mean absorbed tumor dose of 3.5 Gy and 7 Gy, respectively. The tumor sizes and body weights were measured every other day over a period of 70 days.

***Results:*** The tumor size of untreated control mice (Group A) was constantly increasing over time (Fig. S6a). Application of 5 MBq [^177^Lu]Lu-DOTA-folate (Group B) to obtain a tumor dose of 3.5 Gy had only a minor effect on the tumor growth visible by TGDI_2_ and TGDI_5_ values that were 10–20% higher than for untreated control mice (Table S2). Moreover, the tumor growth curve and survival curve of mice of Group B were not significantly different from those of the control group. Administration of 10 MBq [^177^Lu]Lu-DOTA-folate (Group C) to reach a mean absorbed tumor dose of 7 Gy delayed the tumor growth significantly. The TGDIs were increased by 40–100% and the median survival was not reached within the 70 days lasting study, which was in clear contrast to the median survival time of mice of Group B (36 days) and control mice of Group A (26 days), respectively (Table S2, Fig. S6b).


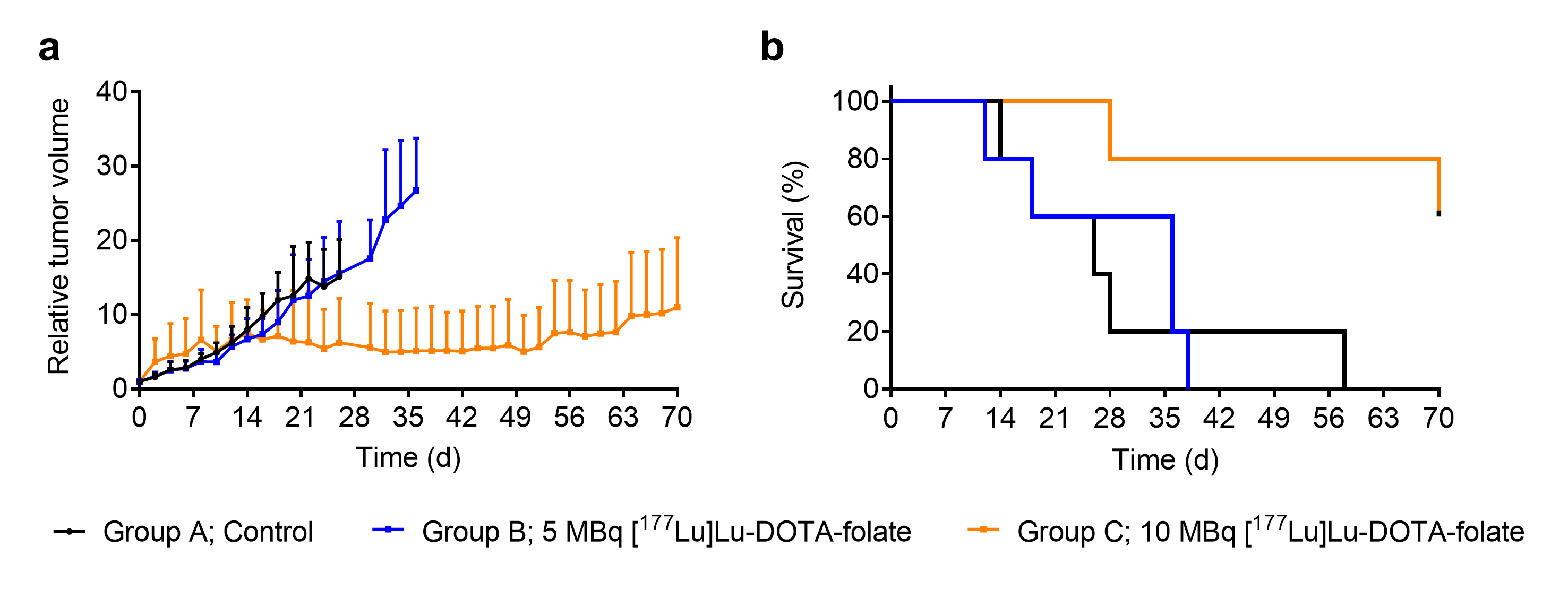


**Fig. S6** NF9006 tumor response to variable quantities of [^177^Lu]Lu-DOTA-folate. (**a**) Tumor growth curves relative to the tumor volume at day 0 (set as 1) for mice that received PBS (Group A), mice treated with 5 MBq [^177^Lu]Lu-DOTA-folate (3.5 Gy tumor dose; Group B) and 10 MBq [^177^Lu]Lu-DOTA-folate (7.0 Gy tumor dose; Group C). (**b**) Kaplan-Meier plot indicating survival curves of mice of Groups A–C.

Table S2 Comparison of the time periods during which mice reached an endpoint, median survival and survival curves as well as tumor growth delay indices (TGDI_x_)

| **Group** | | **Treatment** | **Time frame of euthanasia**  (days) | **Median survival**  (days) | **Survival curve sign. different from groups^1^** | **TGDI_2_** | **TGDI_5_** |
| --- | --- | --- | --- | --- | --- | --- | --- |
| A | Sham | | 14–58 | 26 | C | 1.0 ± 0.6 | 1.0 ± 0.3 |
| B | [^177^Lu]Lu-DOTA-folate , 5 MBq | | 12–38 | 36 | C | 1.2 ± 0.8 | 1.1 ± 0.4 |
| C | [^177^Lu]Lu-DOTA-folate , 10 MBq | | 28–70* | n.d. | A, B | 1.4 ± 1.3 | 2.0 ± 2.4 |

* Day 70 = end of the study

^1^ Comparison of survival curves by log-rank (Mantel-Cox) test

n.d. = not determined

**11. Therapy study**

***Purpose:*** The aim of the therapy study was to investigate the potential of [^177^Lu]Lu-DOTA-folate to sensitize NF9006 tumors to CTLA-4-based immunotherapy.

***Methods:*** The therapy study was performed in two independent experiments with four groups of NF9006 tumor-bearing mice (n=5 and n=6). [^177^Lu]Lu-DOTA-folate was diluted and injected as described in the main article. The injection solutions of the antibodies (200 μg/100 μL) were prepared in a recommended dilution buffer (InVivoPure, pH 7.0, BioXCell). The treatment groups and application schedule are described in the main article (Table 1).

The tumor dimension was determined by measuring the longest tumor axis (L) and its perpendicular axis (W) using a digital caliper to determine the tumor volume (TV) according to the equation [TV = 0.5 × (L × W^2^)]. The relative tumor volume (RTV) was defined as [TV_x_/TV_0_], where TV_x_ is the tumor volume in mm^3^ at a given day x and TV_0_ the tumor volume in mm^3^ at day 0. The relative body weight (RBW) was defined as [BW_x_/ BW_0_], where BW_x_ is the body weight in gram at a given day x and BW_0_ the body weight in gram at day 0. The endpoint criteria are reported in the main article.

For data analysis, one mouse of Group B was excluded because it did not develop a tumor, and one mouse of Group C was excluded due to the premature death after i.p. administration of the antibody.

***Results:*** The results of the therapy study are reported in the main article.

**12. Therapy study: Assessment of the efficacy**

***Purpose:*** In order to quantify the efficacy of the therapy, tumor growth delay indices (TGDIs) were determined among other parameters.

***Methods:*** Tumor growth delay indices were calculated for each individual mouse as described in the main article.

***Results:*** The tumor delay indices are reported in the main article (Fig. 5e) and summarized in Table S3. The values of the TGDI_2_, TGDI_5_ and TGDI_8_ of Group D are lower than it would be if the study was not terminated 70 days after therapy start, as three mice of this group did not reach the RTV of 2 and six mice did not reach a RTV of 5 and 8 during the time of investigation.

**Table S3** Tumor growth delay indices with x-fold increase of tumor size (TGDI_x_) of mice that received only [^177^Lu]Lu-DOTA-folate, anti-CTLA-4 mAb and combination of both agents

| **Group** | **Treatment** | **TGDI_2_** | **TGDI_5_** | **TGDI_8_** |
| --- | --- | --- | --- | --- |
| A | Sham | 1.0 ± 0.3 (n=11) | 1.0 ± 0.3 (n=11) | 1.0 ± 0.3 (n=8) |
| B | [^177^Lu]Lu-DOTA-folate (5 MBq) | 1.1 ± 0.7 (n=10) | 1.2 ± 0.5 (n=10) | 1.2 ± 0.4 (n=9) |
| C | Anti-CTLA-4 mAb | 0.9 ± 0.4 (n=10) | 1.4 ± 0.5 (n=8)  n.d. (n=1)^1^ | 1.4 ± 0.6 (n=7)  n.d. (n=1)^1^ |
| D | Combination | 1.8 ± 1.9 (n=8)  n.d. (n=3)^1^ | 1.8 ± 1.3 (n=4)  n.d. (n=6)^1^ | 1.9 ± 1.1 (n=4)  n.d. (n=6)^1^ |

^1^ TGDI_2_, TGDI_5_ and TGDI_8_ were not determined for these mice due the small RTV which was below 2, 5 or 8, respectively.

**13. Assessment of potential early side effects after therapy**

***Purpose:*** As a measure of early side effects, the body weight of each individual mouse was monitored over the whole period of investigation. When an endpoint was reached, organ mass and mass ratios as well as blood plasma parameters were assessed.

***Methods:*** *Monitoring of the body weights:* The body weight of each individual mouse was measured every other day until an endpoint was reached. Comparison of the average relative body weights (RBW) and absolute body weights (ABW) of control mice and mice of Groups B–D was performed at day 8 and at the endpoint. Body weight data were analyzed for significance using a one-way ANOVA test with a Dunnett’s multiple comparisons post-test (GraphPad Prism software, version 7). A *p* value of <0.05 was considered as statistically significant.

*Blood plasma chemistry:* Immediately before euthanasia of the mice that reached the endpoint, the blood was sampled from the retrobulbar vein. The values of creatinine (CRE), blood urea nitrogen (BUN), alkaline phosphatase (ALP), total bilirubin (TBIL) and albumin (ALB) were determined in blood plasma after centrifugation of the blood using a dry chemistry analyzer (DRI-CHEM 4000i, FUJIFILM, Japan). The average blood plasma parameters of each group were analyzed for significance using a one-way ANOVA test with a Dunnett’s multiple comparisons post-test (GraphPad Prism software, version 7). A *p* value of <0.05 was considered as statistically significant.
*Organ mass and mass ratios:* After euthanasia, selected organs were collected and weighed. The organ mass-to-brain mass ratios were calculated as previously reported [5]. Organ data were analyzed for significance using a one-way ANOVA test with a Dunnett’s multiple comparisons post-test (GraphPad Prism software, version 7). A *p* value of <0.05 was considered as statistically significant.

***Results:*** *Monitoring of the body weights:* No obvious differences in body weights of mice of each group were determined among the single groups (Fig. S7).

**
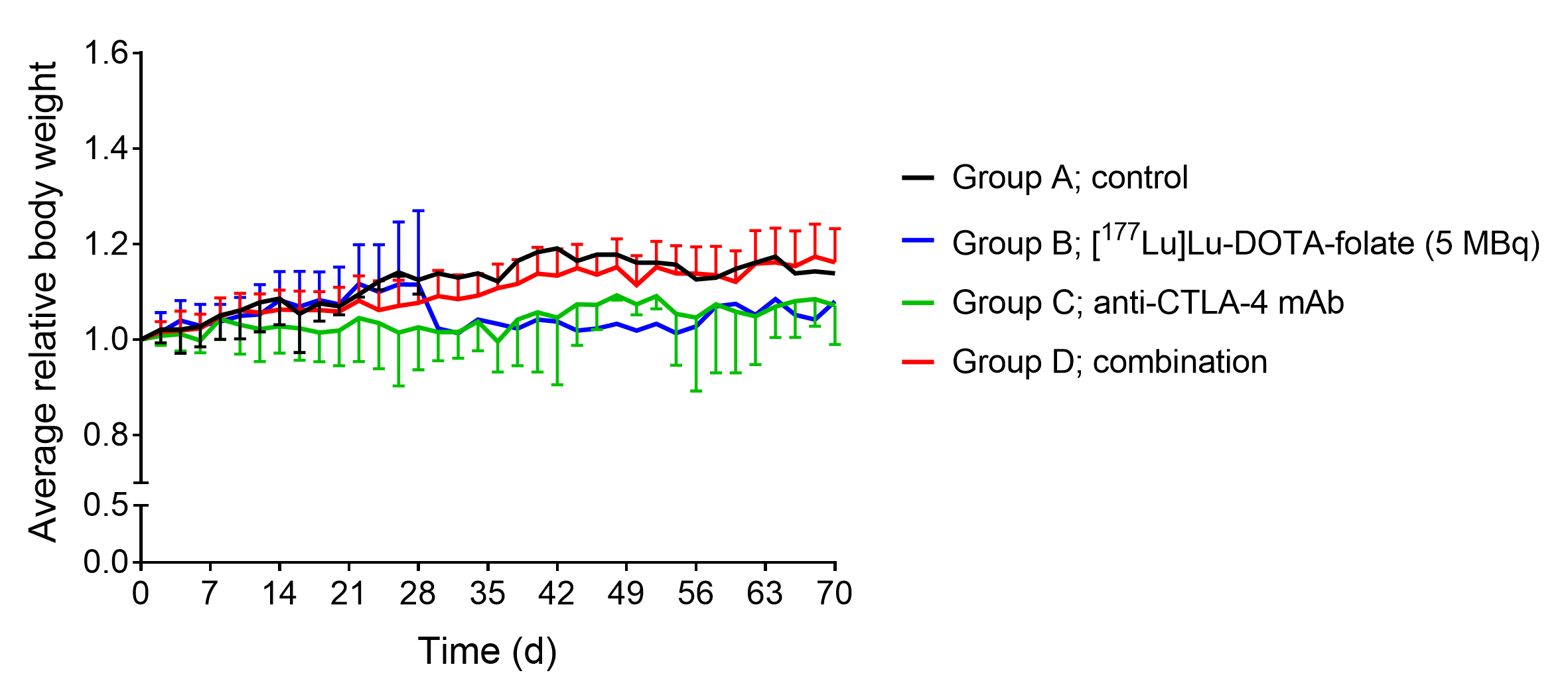
**

**Fig. S7** Relative body weights of mice of the therapy study. Graph showing relative body weights of mice expressed as an average ± SD (for n ≥ 2) of each group in the time course of the study.

At day 8, when the first mouse of the control group reached an endpoint, the body weights of mice of each group were statistically not different from each other (Table S4). Comparison of the relative body weights at the endpoint of the therapy revealed significantly higher values for mice of Group D than for mice of Group A. This may be related to the fact that endpoints of mice of Group D were reached later than for mice of group A.

**Table S4** Relative and absolute body weights of mice at the day of euthanasia of the first control mouse (day 8) and when an endpoint of the therapy study was reached

| **Group** | **Relative body weights at day 8** | **Absolute body weights** **at day 8** (g)  (average ± SD) | **Relative body weights at endpoint or study end**  (average ± SD) | **Absolute body weights at endpoint or study end** (g)  (average ± SD) |
| --- | --- | --- | --- | --- |
| A | 1.05 ± 0.05 | 23.4 ± 2.0 | 1.08 ± 0.05 | 23.9 ± 1.9 |
| B | 1.04 ± 0.04 | 22.1 ± 1.4 | 1.11 ± 0.07 | 23.6 ± 1.3 |
| C | 1.04 ± 0.04 | 22.1 ± 1.6 | 1.05 ± 0.07 | 22.3 ± 1.4 |
| D | 1.05 ± 0.04 | 22.5 ± 1.9 | 1.15 ± 0.07* | 24.7 ± 2.6 |

* Significantly different from the value of control group (Group A) (*p*<0.05)

*Blood plasma chemistry:* No significant differences between the untreated (Group A) and treated mice (Group B, C and D) were determined in blood plasma levels of CRE, BUN, ALP and TBIL at the endpoint of the therapy (Table S5). Only the ALB plasma levels of Group D were elevated compared to the control mice, which might be related to the age difference of both groups at the time when they were euthanized.

Table S5 Plasma chemistry determined at the endpoint of the therapy study

| Group | **CRE^1^**  (µmol/L) | **BUN^1^**  (mmol/L) | **ALP^1^**  (U/L) | **TBIL^1^**  (µmol/L) | **ALB**  (g/L) |
| --- | --- | --- | --- | --- | --- |
| A | < 18 (n=11) | 5.88 ± 0.98 (n=11) | 68 ± 6 (n=11) | < 3 (n=9); 4 ± 1 (n=2) | 22 ± 2 (n=11) |
| B | < 18 (n=10) | 6.69 ± 1.16 (n=10) | 66 ± 9 (n=10) | < 3 (n=9); 3 (n=1) | 23 ± 1 (n=10) |
| C | < 18 (n=9); 21 (n=1) | 6.43 ± 1.23 (n=10) | 71 ± 14 (n=10) | < 3 (n=9); 3 (n=1) | 23 ± 2 (n=10) |
| D | < 18 (n=10); 18 (n=1) | 6.67 ± 1.33 (n=11) | 76 ± 13 (n=11) | < 3 (n=8); 3 ± 0 (n=3) | 24 ± 2* (n=11) |

^1^ No significant differences determined in comparison to the control group (Group A) (*p*>0.05);

* Significantly different from the value of control group (Group A) (*p*<0.05)

*Organ mass and mass ratios:* No statistically significant differences in organ masses and ratios thereof were observed (*p*>0.05) (Table S6).

**Table S6** Organ mass and corresponding organ-to-brain mass ratios obtained for the untreated and treated mice of the therapy study

| **Group**  (n=10–11) | **Organ mass^1^ (mg)**  **(average ± SD)** | | | | **Organ-to-brain mass ratios**  **(average ± SD)** | | |
| --- | --- | --- | --- | --- | --- | --- | --- |
|  | Kidneys | Liver | Spleen | Brain | Kidney-to-brain | Liver-to-brain | Spleen-to-brain |
| A | 274 ± 27 | 1050 ± 80 | 145 ± 39 | 448 ± 20 | 0.61 ± 0.04 | 2.34 ± 0.16 | 0.32 ± 0.09 |
| B | 270 ± 21 | 1125 ± 109 | 118 ± 19 | 443 ± 17 | 0.61 ± 0.04 | 2.54 ± 0.24 | 0.27 ± 0.05 |
| C | 272 ± 22 | 998 ± 73 | 142 ± 56 | 436 ± 17 | 0.63 ± 0.05 | 2.29 ± 0.13 | 0.33 ± 0.14 |
| D | 270 ± 22 | 1074 ± 94 | 112 ± 36 | 448 ± 14 | 0.60 ± 0.04 | 2.40 ± 0.22 | 0.25 ± 0.08 |

^1^ Data obtained at the day of euthanasia when an endpoint criterion was reached or at the end of the study (day 70).

**14. Cell uptake of [^177^Lu]Lu-DOTA-folate in NF9006 and KB cells**

***Purpose:*** NF9006 tumor cell uptake and internalization of [^177^Lu]Lu-DOTA-folate using variable molar amounts of DOTA-folate was determined to better understand potential saturation effect of the FRs by non-radioactive folate due to the lower FR expression level on these cells. For comparison, the same experiments were also performed with KB tumor cells.

***Methods:*** Uptake and internalization of [^177^Lu]Lu-DOTA-folate was determined as previously reported [2], using variable amounts of DOTA-folate (0.75, 1.5, 7.5, 15, 37.5 and 75 pmol per well) while keeping the activity constant. The plates were incubated at 37 °C, 5% CO_2_ for 4 h and afterwards washed according to the same procedure as described above.

***Results:*** For NF9006 cells, application of increasing amounts of DOTA-folate (0.75, 1.5, 7.5 pmol) resulted in decreasing uptake of 50 ± 3%, 27 ± 7% and 6.5 ± 1.1% of [^177^Lu]Lu-DOTA-folate, respectively (Fig. S8a). Using an even higher molar amount of DOTA-folate (15, 37.5, 75 pmol) resulted in almost entire blockade of the FRs and <4% uptake of [^177^Lu]Lu-DOTA-folate in NF9006 cells. The internalized fraction was approximately 50% of the total bound [^177^Lu]Lu-DOTA-folate in each case. In KB cells, the use of [^177^Lu]Lu-DOTA-folate up to 37.5 pmol did not affect the uptake which ranged between 30–39%, whereof the internalized fraction was 12–19% (Fig. S8b). Cells incubated with the highest molar amount of DOTA-folate showed somewhat lower uptake of ~20% and ~7% accounted for the internalized fraction.

***
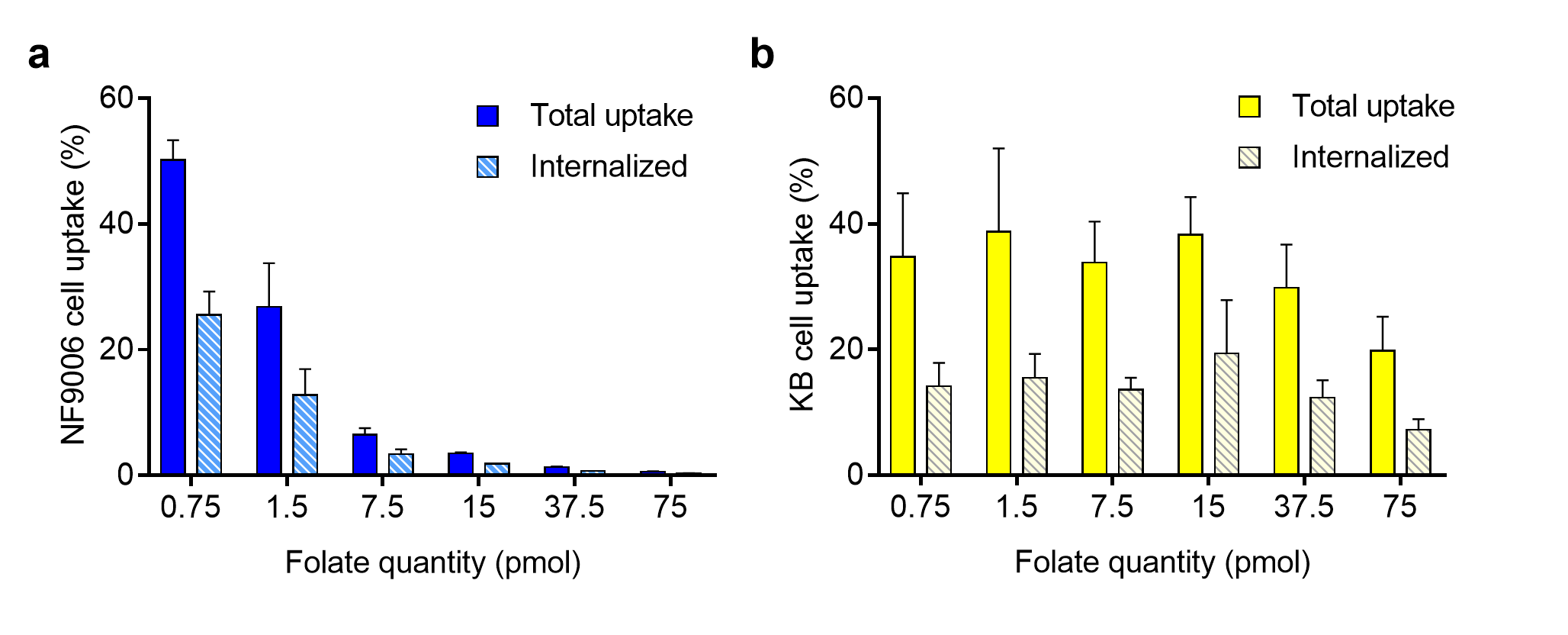
***

**Fig. S8** Cell uptake of [^177^Lu]Lu-DOTA-folate into NF9006 and KB tumor cells using variable molar quantities (indicated as “folate quantity”). (**a**) Total uptake and internalization of [^177^Lu]Lu-DOTA-folate into NF9006 cells; (**b**) Total uptake and internalization of [^177^Lu]Lu-DOTA-folate into KB cells. The results are expressed as average ± SD (n=2–4).

**15. Therapy study: Correlation of initial tumor size with therapeutic efficacy**

***Purpose:*** The aim was to determine on whether or not the efficacy of the therapy was dependent on initial tumor size.

***Methods:*** The therapeutic effect, quantified by TGDIs and survival of each individual mouse, was correlated with the initial tumor size of the respective mouse.

***Results:*** No correlation was observed between the therapeutic efficacy (TGDIs and survival) and the initial tumor size of individual mice as demonstrated in respective graphs (Fig. S9).


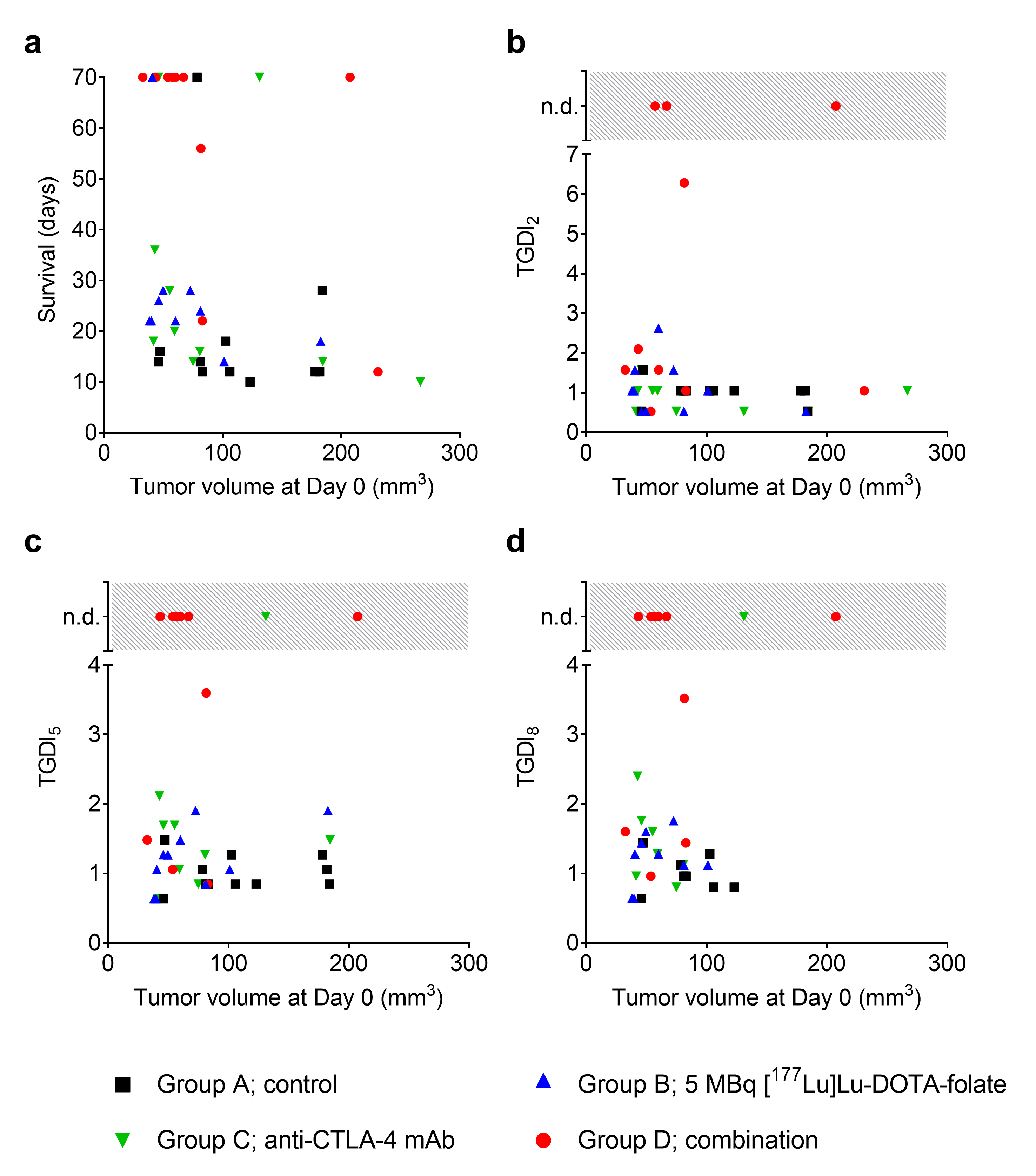


**Fig. S9** Additional data of the therapy study performed with [^177^Lu]Lu-DOTA-folate and anti-CTLA-4 mAb in NF9006 tumor-bearing mice. (**a–d**) Graphs that correlated the initial tumor volume of individual mice and the effect of applied treatment, expressed by (**a**) survival, (**b**) TGDI_2_, (**c**) TGDI_5_, (**d**) TGDI_8_. The TGDI_x_ points marked in the grey filled areas were not determined due to significantly delayed tumor growth, hence, the TGDI_x_ values would be above the shown range.

**16. Assessment of PET radiotracers for monitoring NF9006 tumors**

***Purpose:*** Biodistribution and imaging studies were performed using two positron emission tomography (PET) radiotracers, which may potentially serve for monitoring purposes of the NF9006 tumor model. Among those was ^18^F-AzaFol, a FR-targeted PET radiotracer, which was previously developed in our department and recently tested in a clinical trial [6, 7] and 2-deoxy-2-[^18^F]fluoro-d-glucose ([^18^F]FDG), the most commonly used PET radiotracer in nuclear medicine [8].

***Methods:*** *Radiotracers:* ^18^F-AzaFol was synthesized at ETH Zurich according to a previously reported method [6]. [^18^F]FDG was purchased from the radiopharmacy of the University Hospital Zurich, Zurich.

*Biodistribution studies:* FVB/NCrl mice were subcutaneously inoculated with NF9006 tumor cells
(2.5 × 10^6^ cells) on the right and left shoulder, about two weeks before starting the experiment. Biodistribution studies were performed in quadruplicates, when the tumor size reached a volume of ~100–300 mm^3^. Mice were intravenously injected with ^18^F-AzaFol (5 MBq, 100 µL) or [^18^F]FDG
(5 MBq, 100 µL). Receptor-specific uptake of ^18^F-AzaFol was confirmed by injection of excess folic acid (100 µg, 100 µL per mouse in PBS) to block FRs. The animals were sacrificed at 1 h and 2 h after administration of the respective PET agent. Selected tissues and organs were collected and weighed followed by the measurement of activity in a γ-counter (Perkin Elmer, Wallac Wizard 1480). The results are listed as percentage of the injected activity per gram of tissue mass (% IA/g), using counts of
a defined volume of the original injection solution measured at the same time resulting in decay-corrected values.

*PET/CT imaging:* For PET imaging, FVB/NCrl mice were subcutaneously inoculated with NF9006 tumor cells (2.5 × 10^6^ cells) on the right shoulder, about two weeks before the experiment. PET/CT scans were performed using a small-animal bench-top PET/CT scanner (G8, Perkin Elmer, Massachusetts, U.S [9]), as previously reported, with a set energy window ranging from 150 keV to 650 keV [10]. Mice were intravenously injected with ^18^F-AzaFol (5 MBq/100 µL) or [^18^F]FDG (5 MBq,
100 µL). Static whole-body PET scans of 10 min duration were performed at 1 h and 2 h after injection of the respective PET agent, followed by a CT scan of 1.5 min. During the scan, mice were anesthetized with a mixture of isoflurane and oxygen. The acquisition of the data and their reconstruction was performed using the G8 PET/CT scanner software (version 2.0.0.10). All images were prepared using VivoQuant post-processing software (version 3.5, inviCRO Imaging Services and Software, Boston U.S.). The scale of activity for fluorine-18 was set as indicated on the images.

***Results:*** *Biodistribution studies:* ^18^F-AzaFol showed high accumulation in NF9006 tumors (~14% IA/g, 1 h and 2 h p.i.), but also considerable accumulation in the kidneys (42 ± 4% IA/g at 1 h p.i.; 40 ± 3% IA/g at 2 h p.i.). These findings were in line with previous experiments performed with ^18^F-AzaFol in KB tumor-bearing nude mice [6]. Blockade of the FRs with folic acid reduced the uptake in the tumors and kidneys to <1% IA/g and ~5% IA/g, respectively, indicating FR-specific accumulation in these tissues (Table S7, Fig. S10). Uptake of [^18^F]FDG in NF9006 tumors was low, in the range of ~4% IA/g (Table S7, Fig. S10). As expected, [^18^F]FDG accumulated in the heart, where it was ~8- to 10-fold higher than in the NF9006 tumor.

Due to the high tumor uptake, ^18^F-AzaFol showed higher tumor-to-blood ratios (32 ± 2; 1 h p.i.) than [^18^F]FDG, which reached a value of ~9 at the same timepoint. The tumor-to-liver and tumor-to-kidney ratios of ^18^F-AzaFol were, however, lower than the respective ratios of [^18^F]FDG, due to the lower accumulation of [^18^F]FDG in the liver and kidneys (Table S7).

**Table S7** Biodistribution data of ^18^F-AzaFol and [^18^F]FDG in NF9006 tumor-bearing mice at 1 h and
2 h after injection of the PET agents. The data are decay-corrected and listed as percentage of injected activity per gram tissue (% IA/g), reported as average ± SD obtained from each group of mice (n=4)

|  | **^18^F-AzaFol** | | | **^18^F-FDG** | |
| --- | --- | --- | --- | --- | --- |
|  | **1 h p.i.** | **2 h p.i.** | **1 h p.i. + blockade** | **1 h p.i.** | **2 h p.i.** |
| Blood | 0.45 ± 0.02 | 0.31 ± 0.02 | 0.84 ± 0.19 | 0.44 ± 0.04 | 0.28 ± 0.03 |
| Liver | 8.0 ± 0.2 | 9.3 ± 1.1 | 14 ± 5 | 0.80 ± 0.21 | 0.56 ± 0.11 |
| Kidney | 42 ± 4 | 40 ± 3 | 5.2 ± 1.7 | 1.1 ± 0.1 | 0.80 ± 0.10 |
| Tumor | 14 ± 1 | 14 ± 1 | 0.64 ± 0.18 | 4.1 ± 1.5 | 3.8 ± 0.6 |
| Tu-to-blood | 32 ± 2 | 46 ± 3 | 0.76 ± 0.13 | 9.2 ± 2.8 | 14 ± 3 |
| Tu-to-liver | 1.8 ± 0.1 | 1.5 ± 0.1 | 0.05 ± 0.01 | 5.1 ± 1.3 | 7.1 ± 1.7 |
| Tu-to-kidney | 0.34 ± 0.02 | 0.36 ± 0.03 | 0.13 ± 0.03 | 3.9 ± 1.4 | 5.0 ± 1.1 |


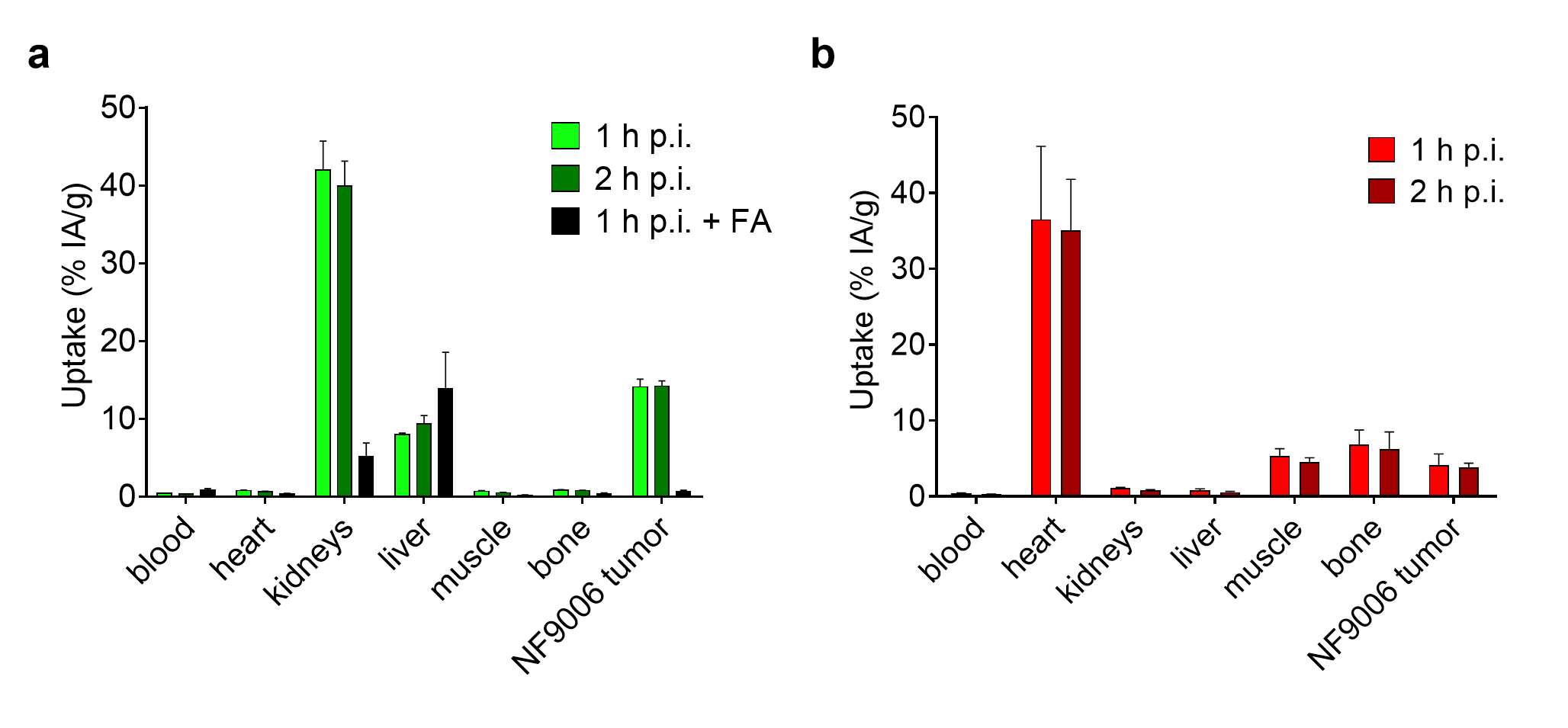


**Fig. S10** Biodistribution data obtained in NF9006 tumor-bearing mice 1 h and 2 h after injection of ^18^F-AzaFol or [^18^F]FDG. (**a**) Tissue distribution profile of ^18^F-AzaFol; (**b**) Tissue distribution profile of [^18^F]FDG. The bars represent the average uptake of injected activity per gram of tissue (% IA/g) ± SD obtained from each group of mice (n=4).

*PET/CT imaging:* PET/CT images confirmed the findings of the biodistribution studies showing distinct accumulation of ^18^F-AzaFol in NF9006 tumors, while uptake of [^18^F]FDG was low and almost not visible on the images (Fig. S11).

**
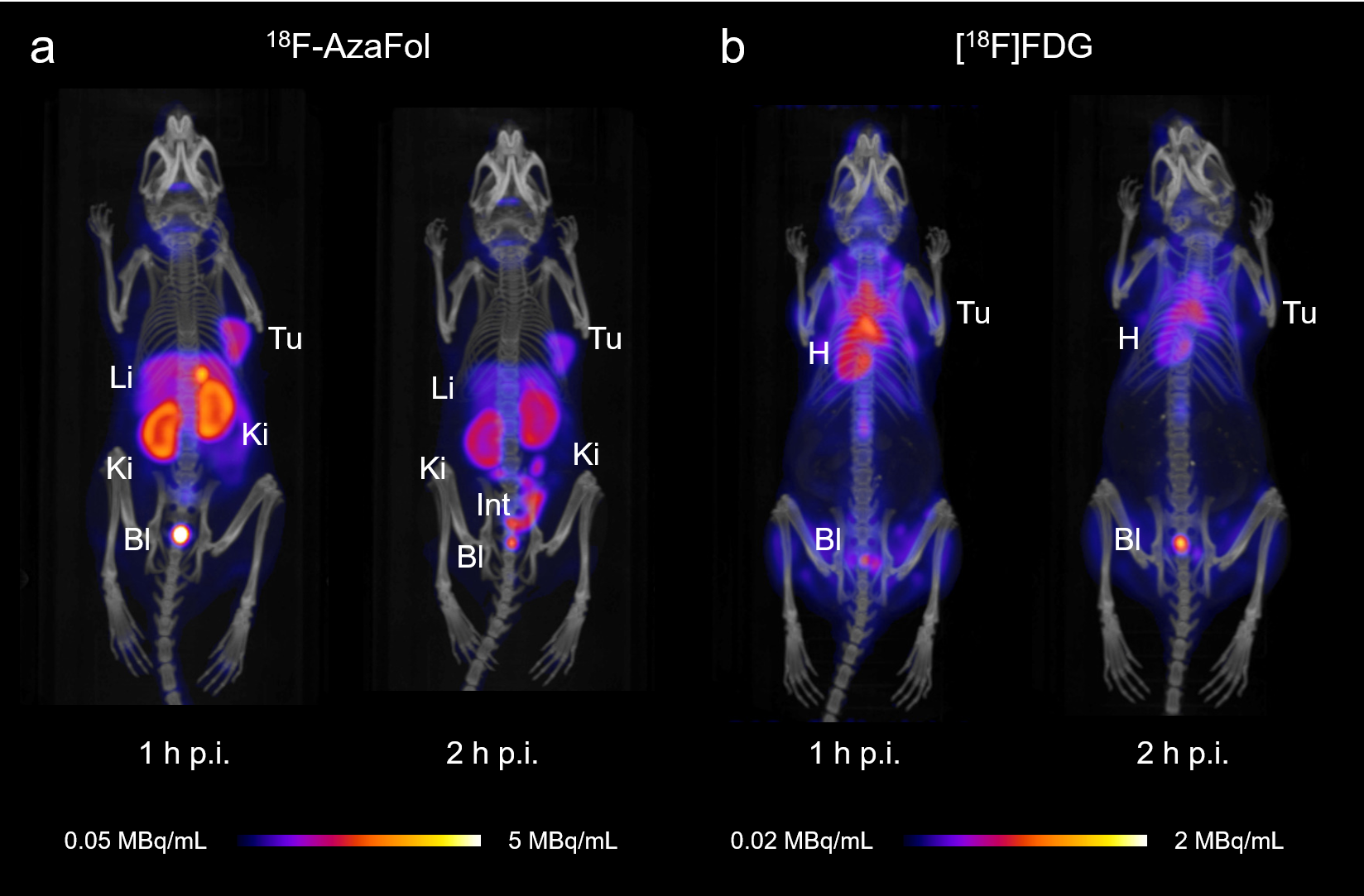
**

**Fig. S11** PET/CT images of NF9006 tumor-bearing mice at 1 h and 2 after injection of or ^18^F-AzaFol or [^18^F]FDG shown as maximum intensity projections (MIPs). (**a**) PET scan obtained with ^18^F-AzaFol;
(**b**) PET scan obtained with [^18^F]FDG.

***Conclusion:*** Based on the high and specific NF9006 tumor uptake and good tumor-to-background ratios, ^18^F-AzaFol revealed to be a suitable PET radiotracer for imaging NF9006 tumors of a syngeneic mouse model. ^18^F-AzaFol may, thus, be used for imaging spontaneous tumors of transgenic FVB/N-Tg(MMTVneu)202Mul/J mice to enable detection of tumors and monitoring therapy response.

**References**

1. Müller C, Struthers H, Winiger C, Zhernosekov K, Schibli R. DOTA conjugate with an albumin-binding entity enables the first folic acid-targeted ^177^Lu-radionuclide tumor therapy in mice. J Nucl Med. 2013;54:124-31. doi:10.2967/jnumed.112.107235.

2. Siwowska K, Haller S, Bortoli F, Benešová M, Groehn V, Bernhardt P, et al. Preclinical comparison of albumin-binding radiofolates: impact of linker entities on the in vitro and in vivo properties. Molecular pharmaceutics. 2017;14:523-32. doi:10.1021/acs.molpharmaceut.6b01010.

3. Müller C, Forrer F, Schibli R, Krenning EP, de Jong M. SPECT study of folate receptor-positive malignant and normal tissues in mice using a novel ^99m^Tc-radiofolate. J Nucl Med. 2008;49:310-7. doi:10.2967/jnumed.107.045856.

4. Salvat F. PENELOPE2014: A code system for Monte-Carlo simulation of electron and photon transport. OECD/NEA Data Bank: NEA/NSC/DOC. 2015;3.

5. Umbricht CA, Benesova M, Schibli R, Müller C. Preclinical development of novel PSMA-targeting radioligands: modulation of albumin-binding properties to improve prostate cancer therapy. Mol Pharm. 2018;15:2297-306. doi:10.1021/acs.molpharmaceut.8b00152.

6. Betzel T, Müller C, Groehn V, Müller A, Reber J, Fischer CR, et al. Radiosynthesis and preclinical evaluation of 3'-aza-2'-[^18^F]fluorofolic acid: a novel PET radiotracer for folate receptor targeting. Bioconjugate Chem. 2013;24:205-14. doi:10.1021/bc300483a.

7. Gnesin S, Müller J, Burger IA, Meisel A, Siano M, Früh M, et al. Radiation dosimetry of ^18^F-AzaFol: A first in-human use of a folate receptor PET tracer. EJNMMI Res. 2020;10:32. doi:10.1186/s13550-020-00624-2.

8. Almuhaideb A, Papathanasiou N, Bomanji J. ^18^F-FDG PET/CT imaging in oncology. Ann Saudi Med. 2011;31:3-13. doi:10.4103/0256-4947.75771.

9. Gu Z, Taschereau R, Vu NT, Prout DL, Silverman RW, Lee JT, et al. Performance evaluation of G8, a high-sensitivity benchtop preclinical PET/CT tomograph. J Nucl Med. 2019;60:142-9. doi:10.2967/jnumed.118.208827.

10. Farkas R, Siwowska K, Ametamey SM, Schibli R, van der Meulen NP, Müller C. ^64^Cu- and ^68^Ga-based PET imaging of folate receptor-positive tumors: development and evaluation of an albumin-binding NODAGA-folate. Mol Pharm. 2016;13:1979-87. doi:10.1021/acs.molpharmaceut.6b00143.
